# Transcriptome Analysis of JA Signal Transduction, Transcription Factors, and Monoterpene Biosynthesis Pathway in Response to Methyl Jasmonate Elicitation in *Mentha canadensis* L.

**DOI:** 10.3390/ijms19082364

**Published:** 2018-08-10

**Authors:** Xiwu Qi, Hailing Fang, Xu Yu, Dongbei Xu, Li Li, Chengyuan Liang, Hongfei Lu, Weilin Li, Yin Chen, Zequn Chen

**Affiliations:** 1 Institute of Botany, Jiangsu Province and Chinese Academy of Sciences, Nanjing 210014, China; qixiwu@126.com (X.Q.); fanghailing2013@163.com (H.F.); yuxu84@163.com (X.Y.); xudongbei2006@126.com (D.X.); xinwenbanlili@163.com (L.L.); ilseyinchen@163.com (Y.C.); zequnchen@126.com (Z.C.); 2 Jiangsu Key Laboratory for the Research and Utilization of Plant Resources, Nanjing 210014, China; 3 School of Environmental and Chemical Engineering, Jiangsu University of Science and Technology, Zhenjiang 212005, China; hongf_lu@163.com; 4 College of Forest, Nanjing Forestry University, Nanjing 210037, China

**Keywords:** *Mentha canadensis* L., transcriptome sequencing, JA signaling, transcription factors, menthol biosynthesis

## Abstract

*Mentha canadensis* L. has important economic value for its abundance in essential oils. Menthol is the main component of *M. canadensis* essential oils, which is certainly the best-known monoterpene for its simple structure and wide applications. However, the regulation of menthol biosynthesis remains elusive in *M. canadensis*. In this study, transcriptome sequencing of *M. canadensis* with MeJA treatment was applied to illustrate the transcriptional regulation of plant secondary metabolites, especially menthol biosynthesis. Six sequencing libraries were constructed including three replicates for both control check (CK) and methyl jasmonate (MeJA) treatment and at least 8 Gb clean bases was produced for each library. After assembly, a total of 81,843 unigenes were obtained with an average length of 724 bp. Functional annotation indicated that 64.55% of unigenes could be annotated in at least one database. Additionally, 4430 differentially expressed genes (DEGs) with 2383 up-regulated and 2047 down-regulated transcripts were identified under MeJA treatment. Kyoto Encyclopedia of Genes and Genomes (KEGG) enrichment indicated that “Monoterpenoid biosynthesis” was one of the most significantly enriched pathways in metabolism. Subsequently, DEGs involved in JA signal transduction, transcription factors, and monoterpene biosynthesis were analyzed. 9 orthologous genes involved in menthol biosynthesis were also identified. This is the first report of a transcriptome study of *M. canadensis* and will facilitate the studies of monoterpene biosynthesis in the genus *Mentha*.

## 1. Introduction

The genus *Mentha* has important economic value for its abundance in essential oils that are widely used in the flavor, fragrance, and aromatherapy industries [1]. Monoterpenes are the major constituents of essential oils, which represent a large and diverse class of volatile C10 isoprenoids. The biosynthesis of monoterpenes originated in the upstream methylerythritol phosphate (MEP) pathway, then catalyzed by a series of enzymes. The catalytic mechanisms of monoterpene biosynthetic enzymes have been extensively studied in peppermint (*Mentha* × *piperita* L.) and spearmint (*Mentha spicata* L.), two well-known *Mentha* plants that have been employed as model systems for the study of monoterpene biosynthesis [2,3,4,5,6,7]. In genus *Mentha*, a variety of monoterpenes including (−)-menthol, (+)-neomenthol, (+)-isomenthol, (+)-carvone, (+)-menthofuran, and so on, are synthesized and stored in the peltate glandular trichomes on the aerial surfaces of the plant [8,9]. The oil compositions vary in different *Mentha* species and are regulated by transcriptional abundance, catalytic properties of enzyme catalysts, and cell type-specific epigenetic processes [9].

*Mentha canadensis* L., as the largest cultivated aromatic plant in the world, is mainly cultivated in China. Not only in the aromatic industry, *M. canadensis* is also widely used as a medicinal plant in traditional Chinese medicine [10]. The main component of *M. canadensis* essential oils is menthol which accounts for more than 78% of the total essential oils’ components [11]. Menthol is certainly the best-known monoterpene for its simple structure and wide applications. The earlier studies of *M. canadensis* mainly focused on the identification of active compounds and activity assays [12,13,14]. Since the absence of sequence data, the molecular mechanism of menthol biosynthesis in *M. canadensis* is still not clear. Only three menthol biosynthetic genes have been cloned in *M. canadensis*, including the Geranyl diphosphate synthase large subunit (GPPS-l), Geranyl diphosphate synthase small subunit (GPPS-s), and (−)-Limonene synthase (LS) [15].

The phytohormone jasmonate (JA) is an important regulator in plant responses to biotic and abiotic stresses as well as other metabolic pathways by extensive reprogramming of gene expression [16,17]. It is also an efficient elicitor of plant secondary metabolite production [18]. A lot of plant active ingredients including artemisinin, vinblastine, nicotine, taxol and ginsenoside may be transcriptional regulated by JA signal [19]. In *Artemisia annua*, high content of artemisinin could be produced in response to JA elicitor [20]. Some JA-responsive transcription factors (TFs) such as AaWRKY1, AaERF1, and AaERF2 could regulate the transcription of artemisinin biosynthetic genes and increase the accumulation of artemisinin [21,22,23]. So far, little is known about JA signal response and its effect on monoterpene biosynthesis in *M. canadensis*.

The transcriptome sequencing technology is a useful method to carry out genome-wide gene discovery and expression profiling for its high-throughput and accuracy. It has been widely used to explore plants’ physiological mechanism at the molecular level, such as model plants *Arabidopsis thaliana* [24] and *Oryza sativa* [25], as well as other non-model plants, such as *Brassica napus* [26], *A. annua* [27], and *Salvia miltiorrhiza* [28]. Using transcriptome sequencing, genome-wide changes in gene expression patterns under different treatment, such as hormone treatment, biotic and abiotic stress, could be easily accessed. For example, JA-mediated transcriptional reprogramming has been studied using RNA-seq in many plants, such as *A. annua* [27], *Taraxacum koksaghyz* [29], and *Gentiana macrophylla* [30].

In this study, high-throughput RNA-seq was applied to analyze the differential gene expression profiles of MeJA-treated *M. canadensis* versus the control. As a result, JA-responsive signal transduction genes, TFs, and monoterpene biosynthetic genes were identified. The results in this study would help us to further understand the reprogramming of JA-responsive gene expression in *M. canadensis* as well as facilitate studies of monoterpene biosynthesis in genus *Mentha*.

## 2. Results

### 2.1. Transcriptome Sequencing and De Novo Assembly of M. canadensis

Transcriptome sequencing was performed for *M. canadensis* under control check (CK) and MeJA treatment using Illumina HiSeq™ 4000. Six sequencing libraries were constructed including three replicates for both CK and MeJA treatment. As a result, each library produced at least 54 Mb clean reads and 8 Gb clean bases. The percentage of clean reads was more than 98% and Q20 percentage was at least 98.69% for the six libraries (Table 1). Then, clean reads of the six libraries were de novo assembled using Trinity program and finally 81,843 unigenes were generated with a GC percentage of 43.92%. The average length and N50 length for the assembled unigenes were 724 and 1126 bp, respectively (Table 2). The length distribution of the unigenes is indicated in Supplementary Figure S1 and 70.63% of all unigenes show lengths longer than 300 bp.

### 2.2. Functional Annotation of Unigenes

For functional annotation analysis, all the assembled unigenes were searched against the public databases using BLAST. The results show that 52,700 (64.39%) unigenes could be annotated in Nr (non-redundant protein) database, 34,565 (42.23%) in Swissprot, 29,536 (36.09%) in KOG (Clusters of eukaryotic Orthologous Group), and 19,013 (23.23%) in KEGG (Kyoto Encyclopedia of Genes and Genomes). Taken together, there were 52,826 (64.55%) unigenes that could be annotated in at least one database (Table 3). GO (Gene Ontology), KOG and KEGG assignments were used to classify the function of the assembled unigenes of *M. canadensis*. For GO annotation, 4396, 4422 and 2566 unigenes were assigned to “biological process” category, “molecular function” category and “cellular component” category, respectively (Supplementary Figure S2). In the “molecular function” category, genes assigned to “binding” (2260) and “catalytic activity” (3326) accounted for the vast majority of this category. In the “biological process” category, “cellular process” (2891), “metabolic processes” (3447) and “single-organism process” (2344) were the main subcategories. For KOG classification, 29,536 unigenes were categorized into 25 KOG functional groups. Among them, “General functional prediction only” (9523) was the largest group, followed by “Signal transduction mechanisms” (5796) and “Posttranslational modification, protein turnover, chaperones” (4901) (Supplementary Figure S3). To further understand the biological functions of *M. canadensis* unigenes, KEGG pathway analysis was performed. As a result, a total of 10,365 unigenes could be mapped to 133 metabolic pathways. “Metabolism” (5104) was the largest category (Supplementary Figure S4).

### 2.3. Identification and Analysis of Differentially Expressed Genes (DEGs)

**Figure 1:**
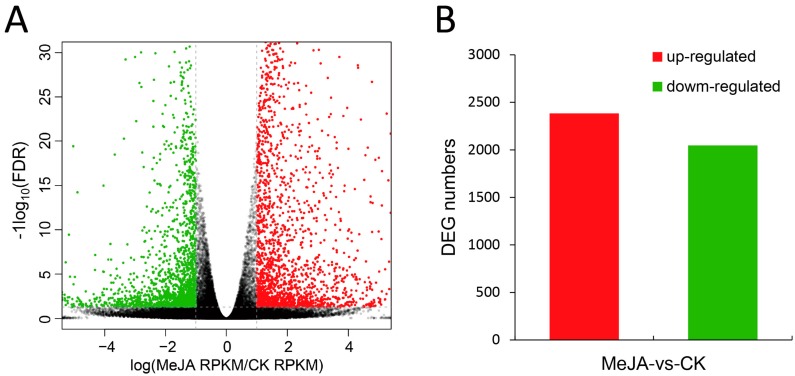
Statistics of DEGs induced by MeJA in *M. canadensis* transcriptomes. (**A**) Volcano plots of the unigenes in the comparisons of MeJA-treated and CK samples; (**B**) DEG numbers in the comparisons of MeJA-treated and CK samples. RPKM: Reads per Kb per Million Reads, FDR: False Discovery Rate, DEG: Differentially Expressed Gene.

**Figure 2:**
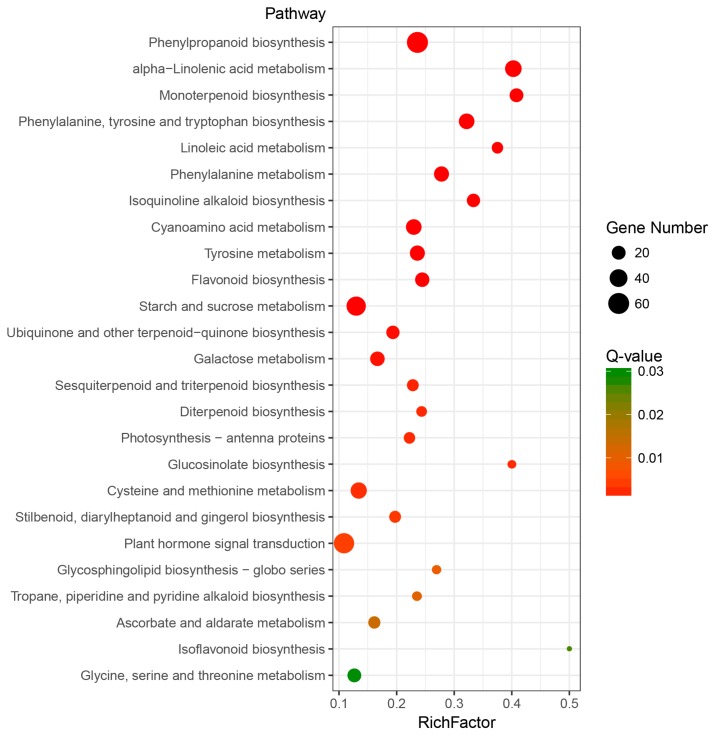
KEGG pathway enrichment of DEGs induced by MeJA.

To identify differentially expressed genes under MeJA treatment, reads were mapped to the unigenes and reads per kb per million reads (RPKM) was used to measure the transcriptional levels. The Pearson correlation analysis indicated that there were high correlations between three biological replicates of both CK and MeJA treatment (Supplementary Figure S5). Then using a two-fold change of RPKM with False Discovery Rate (FDR) <0.05 as the cut-off criteria, 4430 differentially expressed genes (DEGs) were identified between CK and MeJA-treated samples in *M. canadensis*. Among them, 2383 DEGs were up-regulated and 2047 DEGs were down-regulated under MeJA treatment (Figure 1). To further explore the possible roles of the DEGs, GO and KEGG enrichment were conducted. For GO enrichment, 88, 27 and 6 GO terms were significantly enriched in “biological process”, “molecular function” and “cellular component” categories, respectively (Supplementary Table S1). “Terpene synthase activity” and “oxidoreductase activity” were the most significantly enriched terms in “molecular function”. In “biological process” category, biosynthetic processes involved in lipid, small molecule, dicarboxylic acid, phospholipid, terpene and sesquiterpene were the most significantly enriched terms. For KEGG pathway enrichment, 25 pathways were significantly enriched under MeJA treatment (Figure 2 and Supplementary Table S2). Of these, pathways involved in secondary metabolism accounted for a large part, including biosynthetic pathways of phenylpropanoid, terpenoid, alkaloid, flavonoid, glucosinolate and so on.

### 2.4. Identification and Expression Verification of JA Signal Pathway Genes under MeJA Treatment

**Figure 3:**
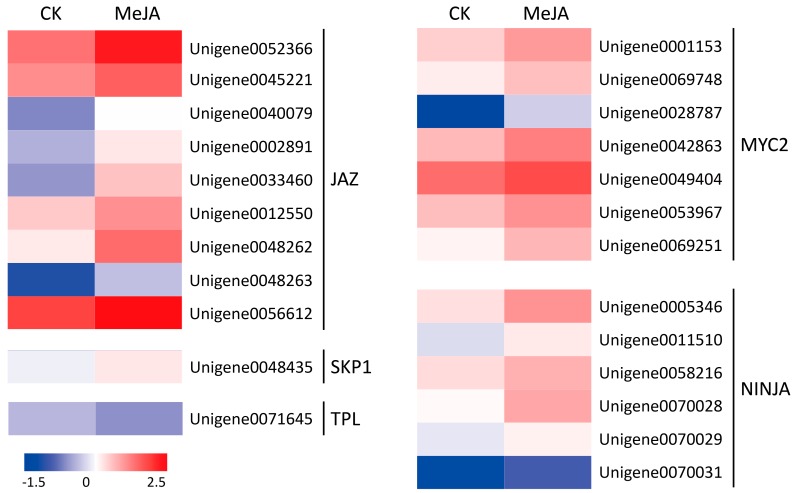
Heat maps of the DEGs in JA signal transduction pathway. JAZ, Jasmonate ZIM domain protein; MYC2, bHLHzip transcription factor MYC2; TPL, TOPLESS; NINJA, Novel interactor of JAZ; SKP1, S-phase kinase-associated protein 1.

**Figure 4:**
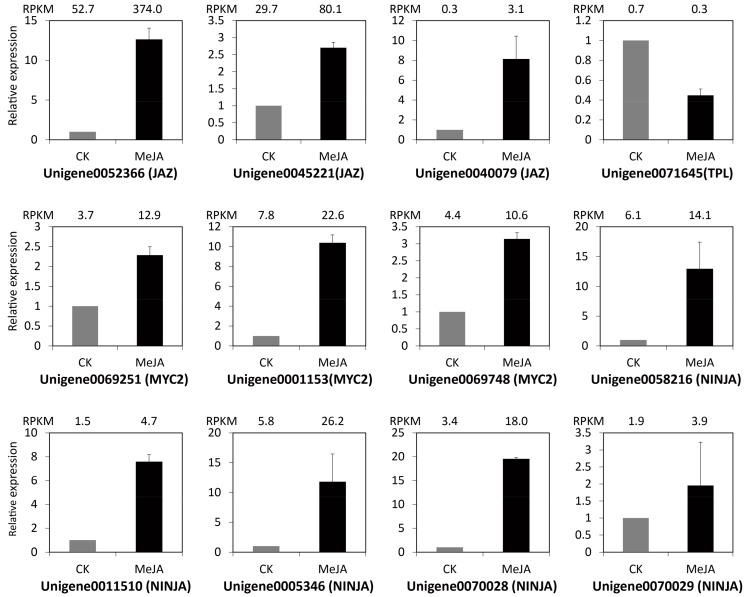
Expression validations of 12 selected JA signal transduction related genes in control and MeJA-treated samples using qRT-PCR. The RPKM values obtained from RNA-Seq data were indicated on the top of each graph.

In Arabidopsis, the JA signal is perceived and transduced via the SCF^COI1^-JAZ co-receptor complex. The SCF^COI1^ complex includes COI1, ASK2, CULLIN1, Rbx, and E2. The JAZ repressors recruit the co-repressor complex consisting of Novel Interactor of JAZ (NINJA), TOPLESS (TPL), and histone deacetylase (HDA), interact with and repress the transcription activator bHLHzip transcription factor MYC2 (MYC2). The perception of JA-Ile by SCF^COI1^ complex leads to degradation of JAZs via the 26S proteasome, then activate the downstream transcription factors of JA responses [17,31]. In this study, 24 DEGs associated with JA signal transduction were identified in *M. canadensis*. Of these, 23 DEGs showed up-regulation under MeJA treatment, which encode putative JAZ (9), MYC2 (7), NINJA (6), and S-phase kinase-associated protein 1 (SKP1) (1) (Figure 3). 12 of the 24 DEGs were selected for quantitative real-time PCR (qRT-PCR) to verify the reliability of the RNA-Seq data. The results showed that all the expression patterns of qRT-PCR were quite a good match with the RPKM results of RNA-Seq data (Figure 4). These validation results of qRT-PCR indicated that the RNA-Seq data were quite reliable.

### 2.5. Identification and Analysis of Differentially Expressed TFs under MeJA Treatment

**Figure 5:**
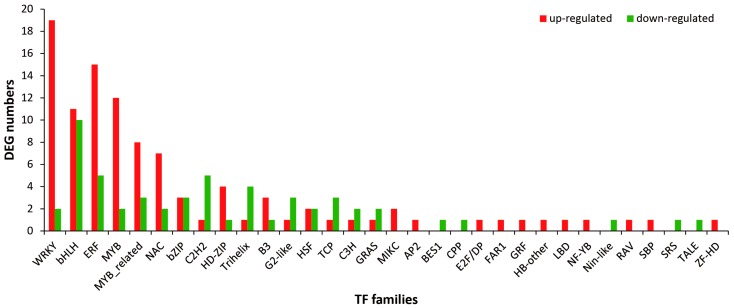
Statistics of differentially expressed TFs under MeJA treatment.

To further understand the transcriptional regulation of MeJA-treated *M. canadensis*, differentially expressed TFs were identified between CK and MeJA-treated samples. As a result, 1535 TFs belonged to 55 families were identified by annotating in PlantTFDB database. bHLH (basic/helix-loop-helix), ERF (ethylene-responsive factor), MYB_related (myeloblastosis DNA-binding related protein), C2H2 (C2H2 zinc-finger protein), MYB (myeloblastosis DNA-binding protein), WRKY (WRKY-type DNA binding protein), C3H (Cys3His zinc finger protein), NAC (NAC domain protein), bZIP (basic region/leucine zipper motif), and GRAS (Gibberellin-insensitive (GAI), Repressor of GA (RGA), and SCARECROW (SCR) protein) families were the top 10 largest TF families (Supplementary Table S3). Of the 1535 TFs, 157 belonged to 32 families were differentially expressed. WRKY, bHLH, ERF, MYB, MYB_related, NAC, bZIP, C2H2, HD-ZIP (Homeodomain-leucine zipper protein), and Trihelix families were the top 10 families that had the largest number of DEGs. Each of these 10 families had more than 5 DEGs and the WRKY and bHLH family were the largest differentially expressed families that both contained 21 DEGs (Figure 5 and Supplementary Table S3). Comparing the expression levels of these DEGs, 102 of the 157 DEGs were up-regulated and the other 55 were down-regulated. Considering the family distribution of the up-regulated and down-regulated DEGs, 19 families showed that the up-regulated unigenes were more abundant than the down-regulated ones, especially in the WRKY family, 19 unigenes were up-regulated and only 2 were down-regulated. On the contrary, there were 11 families that contained more down-regulated unigenes than up-regulated and 2 families contained the same number of up-regulated unigenes and down-regulated (Figure 5).

### 2.6. Identification and Expression Verification of Monoterpenoids and Menthol Biosynthetic Genes under MeJA Treatment

**Figure 6:**
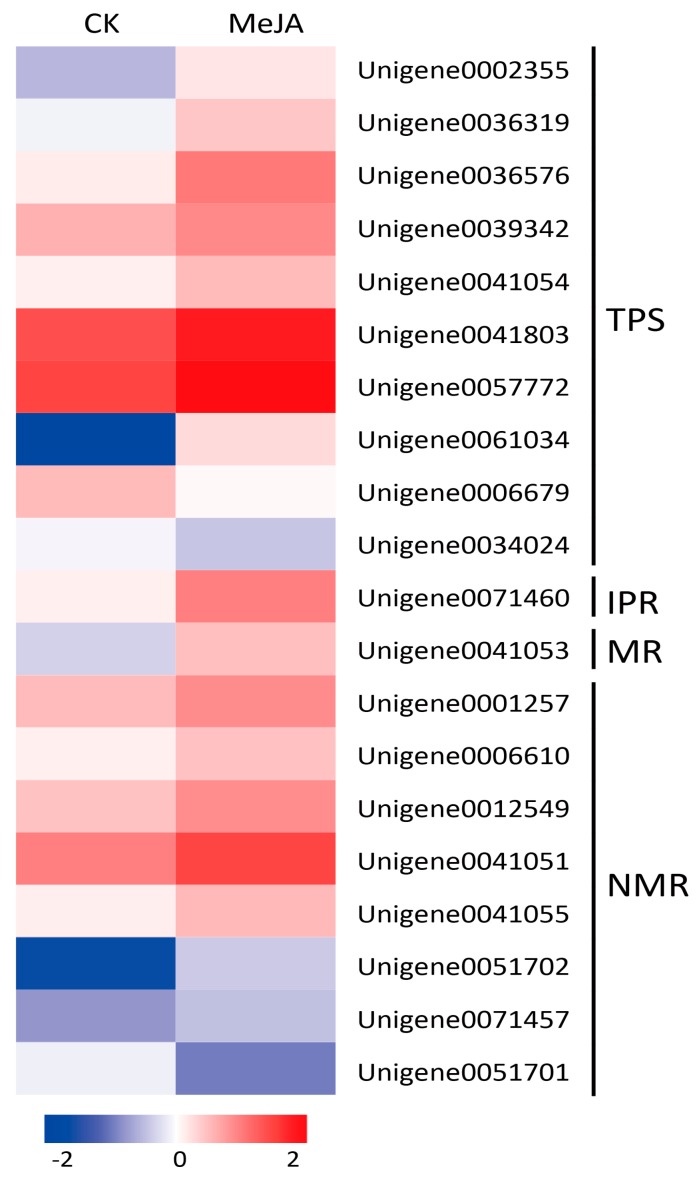
Heat maps of the DEGs in the monoterpenoid biosynthesis pathway. TPS, Terpene synthase; IPR, (−)-Isopiperitenone reductase; MR, (−)-Menthol dehydrogenase; NMR, (+)-Neomenthol dehydrogenase.

In *M. canadensis*, monoterpenes are the major constituents of essential oils. In this study, the enrichment results of the KEGG database indicated that “Monoterpenoid biosynthesis” was one of the most significantly enriched pathways under MeJA treatment. 20 DEGs associated with monoterpenoid biosynthesis were identified, which consisted of genes encoding 10 terpene synthases (TPSs), 1 (−)-isopiperitenone reductase (IPR), 1 menthol dehydrogenase (MR), and 8 neomenthol dehydrogenases (NMRs). *TPS* is a multi-gene family that is widespread in plants, whose diversity and substrate complexity lead to the wide variety of terpenoids [32]. In this study, 10 differentially expressed *TPSs* were identified under MeJA treatment, 8 of which were up-regulated and the other 2 were down-regulated. The remaining 10 DEGs in “Monoterpenoid biosynthesis” were menthol/neomenthol biosynthesis related genes. Interestingly, the expression levels of the 10 DEGs were almost all up-regulated except for an *NMR* (Unigene0051701) under MeJA treatment (Figure 6).

**Figure 7:**
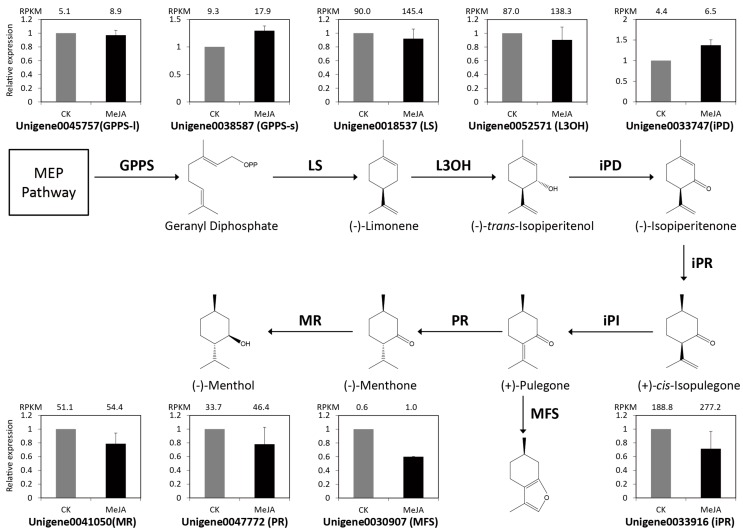
Expression validations of 9 menthol biosynthetic genes in control and MeJA-treated samples using qRT-PCR. The RPKM values obtained from RNA-Seq data are indicated on the top of each graph. GPPS, Geranyl diphosphate synthase; LS, (−)-Limonene synthase; L3OH, (−)-Limonene-3-hydroxylase; iPD, (−)-trans-Isopiperitenol dehydrogenase; iPR, (−)-Isopiperitenone reductase; iPI, (+)-*cis*-Isopulegone isomerase; PR, (+)-Pulegone reductase; MFS, Menthofuran synthase; MR, (−)-Menthol dehydrogenase.

Menthol is the main constituent of monoterpenoids in *M. canadensis*, which is synthesized by a series of enzymatic reactions in the peltate glandular trichomes on the aerial surfaces of the plant. The biosynthetic pathway and enzyme catalysis mechanism of menthol has been extensively studied in peppermint and spearmint, two other species of genus *Mentha* [2,3,4,5,6]. Using the reference genes of peppermint and spearmint as queries, 9 orthologous genes were identified in *M. canadensis* (Supplementary Figure S6), including the previously reported *GPPS-l*, *GPPS-s*, and *LS* [15]. The other 6 genes that catalyze the biosynthesis of (−)-Menthol from (−)-Limonene were identified in *M. canadensis* for the first time. Considering the transcriptional levels of the 9 genes, although their RPKMs showed a certain level of increase after MeJA treatment, they did not reach significant levels above controls. Then, qRT-PCR was used to verify the expression of the 9 menthol biosynthetic genes (Figure 7). The results indicated that the expression levels of most genes were not significantly different between MeJA treatment and CK groups, which was consistent with the RNA-seq results. Among these genes, only Unigene0030907 (*MFS*) showed significant down-regulated expression after MeJA treatment. Unigene0038587 (*GPPS-s*) and Unigene0033747 (*iPD*) showed a certain degree of up-regulation in both qRT-PCR and RNA-Seq data.

## 3. Discussion

Transcriptome sequencing is an effective tool for large-scope gene identification and expression profiling analysis. In this study, high-throughput RNA-seq was applied to characterize the transcriptomes of *M. canadensis* treated with MeJA. Six sequencing libraries were constructed including three replicates for both CK and MeJA treatment and at least 8 Gb clean data was produced for each library. After assembly, a total of 81,843 unigenes were obtained with an average length of 724 bp. Functional annotation indicated that 64.55% of unigenes could be annotated in at least one database. This is the first reported transcriptome study of *M. canadensis* and will provide useful information for further study of this species.

Endogenous MeJA is believed to be a primary regulator of the JA signal pathway in plants. In Arabidopsis, the JA signal is perceived and transduced via the SCF^COI1^-JAZ co-receptor complex. In the resting state, JAZ proteins interact with and repress downstream MYC2 to suppress of JA responses. In response to JA signal, JAZs are degraded by SCF^COI1^-ubiquitin-proteasome, then MYC2 is released from the repressor complex to regulate gene expression [17,31]. In this transcriptome dataset, 24 DEGs associated with JA signal transduction were identified in *M. canadensis*. Of these, 23 DEGs showed up-regulation under MeJA treatment, encoding putative JAZ (9), MYC2 (7), TPL (1), NINJA (6), and SKP1 (1). qRT-PCR was also used to verify the reliability of the RNA-Seq data. These results suggested that application of exogenous MeJA may regulate the JA signaling pathway in *M. canadensis*.

TFs are sequence specific DNA-binding proteins that interact with the regulatory regions of the target genes and regulate their expression. Several transcriptome profiling studies have indicated that MeJA treatment triggers an extensive transcriptional reprogramming of metabolism [33,34,35]. A lot of TFs are elicited by MeJA and participate in regulation of specific metabolic processes [36]. Here, 157 differentially expressed TFs (102 up-regulated and 55 down-regulated) belonged to 32 families were identified by annotating in PlantTFDB database. It is worth noting that the WRKY family was the largest differentially expressed family containing 21 DEGs. It is also reported that more than 25% of WRKYs are induced in response to jasmonate in *A. thaliana* and *Catharanthus roseus* [37]. Of the 21 differentially expressed WRKYs, the number of up-regulated genes was much higher than that of down-regulated ones (19 vs. 2). Similar observations have been found in other plants. For example, 16 up-regulated and 3 down-regulated WRKY TFs were identified under MeJA treatment in *A. annua* [27]. In *Lycoris aurea*, 32 differentially expressed WRKY TFs were identified, of which 26 were up-regulated and only 6 were down-regulated [38]. The WRKY family is an important class of JA-responsive TFs that regulate plant secondary metabolism [39]. In *A. annua*, a WRKY TF *AaWRKY1* was strongly induced by MeJA and regulated the expression of *ADS*, a key gene of artemisinin biosynthesis [21]. In *Panax quinquefolius*, a MeJA-responsive WRKY TF *PqWRKY1* was isolated, which could positively regulate the biosynthesis of triterpene ginsenoside [40]. The large number of up-regulated WRKY TFs in *M. canadensis* suggests that they may function in JA-responsive transcriptional regulation of secondary metabolism.

Monoterpenes are the major constituents of *M. canadensis* essential oils. In this study, the KEGG pathway enrichment of DEGs indicated that “Monoterpenoid biosynthesis” was the most significantly enriched pathway under the treatment of MeJA. 20 DEGs associated with monoterpenoid biosynthesis were identified, which consisted of genes encoding TPS (10), IPR (1), MR (1), and NMR (8). Of these, genes encoding IPR, MR, and NMR were involved in menthol and neomenthol biosynthesis and interestingly, the expression levels of the 10 DEGs were almost all up-regulated under MeJA treatment. These results suggest that the biosynthesis of monoterpenes including menthol and neomenthol might be elicited by the MeJA signal in *M. canadensis*. The biosynthesis pathway of menthol has been extensively studied in peppermint and spearmint [2,3,4,5,6]. In this study, 9 orthologous genes were identified in *M. canadensis* including 6 new reported genes. However, expression analysis of these 9 genes in CK and MeJA-treated samples indicated that most of the gene expression levels did not change significantly after treatment. qRT-PCR results indicated that *GPPS-s* and *iPD* showed a certain degree of up-regulation and *MFS* was down-regulated. We speculate that only part of the menthol biosynthetic genes responds to the JA signal in *M. canadensis.* The response mechanism of menthol biosynthetic genes to JA signaling needs further study in *M. canadensis*, especially the possible DEGs including *GPPS-s*, *iPD*, and *MFS*. Another explanation might be that different secondary metabolic pathways are controlled by different regulatory modules. For example, in *Medicago truncatula*, genes involved in phenylpropanoid biosynthesis were transiently induced after application of MeJA at low concentrations (0.5–5 μmol/L), but triterpene biosynthetic genes were induced after 12–24 h at high concentrations (5–500 μmol/L) [41]. Many *M. canadensis* menthol biosynthetic genes might not respond to the MeJA signal under our treatment conditions (200 μmol/L, 24 h).

Transcriptional regulation is central to plant secondary metabolism. Compared with well-studied metabolic pathways such as flavonoids, the transcriptional regulation of terpene metabolism has been validated in a few studies. Several TFs have been identified from plants including *A. annua* (AaWRKY1, AaERF1, AaERF2, AabZIP1, and AaMYC2), *Gossypium arboretum* (GaWRKY1), *Taxus chinensis* (TaWRKY1), *Hevea brasiliensis* (EREBP1 and HbWRKY1), and *O. sativa* (OsTGAP1) that regulate terpene biosynthesis [21,22,23,42,43,44,45,46,47,48]. In *Mentha* species *M. spicata*, two TFs MsYABBY5 and MsMYB (*M. spicata* MYB DNA-binding protein) have been reported that negatively regulate monoterpene production [49,50]. In this study, homologous genes of the two TFs (Unigene0062650, homolog to *MsYABBY5* and Unigene0030793, homolog to *MsMYB*) were identified in *M. canadensis*. Sequence alignment indicated that both genes had quite high similarities between *M. spicata* and *M. canadensis* (Supplementary Figure S7). Expression patterns deduced from RPKM values indicated that the expression of the two homologous genes declined to a certain extent under MeJA treatment (52.9-vs.-35.6 for Unigene0062650 and 0.72-vs.-0.38 for Unigene0030793), although the decline was not significantly (two-fold change with FDR <0.05). These results suggest that Unigene0062650 and Unigene0030793 might be MeJA-responsive TFs and similar negative regulation mechanism may also exist in *M. canadensis*.

## 4. Materials and Methods

### 4.1. Plant Material and JA Treatment

The *M. canadensis* used for this study was maintained at the Germplasm Nursery in the Institute of Botany, Jiangsu Province and Chinese Academy of Sciences, Nanjing, Jiangsu Province. Mint plants were planted in plastic pots containing a mixture of organic nutrient soil (XingNong Organic Fertilizer Co., Ltd., Zhenjiang, China) and vermiculite (3:1, *v*/*v*). The plants were cultured in an artificial climate chamber (Jiangnan, Ningbo, China) under 14 h light/10 h dark cycles (120 μmol/m^2^/s) with a constant temperature at 25 °C. For MeJA (Aladdin, Shanghai, China) treatment, plants were sprayed with 10 mL 200 μmol/L MeJA that dissolved with 2% ethanol (Sangon, Shanghai, China), and further wrapped in plastic wrap for 24 h to prevent water evaporation. 2% ethanol solution was used as control and the plants were also wrapped in plastic wrap. After 24 h, leaves of mint plants were harvested and frozen in liquid nitrogen and stored at −80 °C. Three biological replicates for both CK and MeJA treatment were performed.

### 4.2. RNA Extraction, cDNA Library Construction and Illumina Sequencing

Total RNA of the *M. canadensis* samples was extracted using RNAiso Plus (Takara, Dalian, China) according to the manufacturer’s instructions. The quality and concentration of RNAs were measured using a ND-2000 UV spectrophotometer (Nanodrop Technologies, Wilmington, DE, USA) and Agilent 2100 (Agilent, Santa Clara, CA, USA). cDNA library construction and Illumina sequencing were performed by Gene Denovo Biotechnology Co. (Guangzhou, China). Briefly, mRNA was enriched from total RNA by Oligo(dT) beads and then fragmented into short fragments. The fragmented mRNA was reverse transcribed into cDNA with random primers and second-strand cDNA was subsequently synthesized. Then the cDNA fragments were purified, end repaired, poly(A) added, and ligated to sequencing adapters. After size selection by agarose gel electrophoresis, the ligation products were PCR amplified and sequenced using Illumina HiSeq^TM^ 4000 (Illumina, San Diego, USA).

### 4.3. Transcriptome Assembly and Annotation

The raw reads obtained from RNA sequencing were filtered by trimming adapters and removing low quality reads (reads containing more than 10% of unknown nucleotides (N) or 40% of low quality (Q-value ≤ 10) bases) to obtain the high-quality clean reads. All data generated in this study have been deposited in the National Center for Biotechnology Information (NCBI) and can be accessed in the Short Read Archive (SRA) Sequence Database under accession number SRP132644. Then, Trinity program was used to perform de novo assembly of the clean data [51]. After assembly, unigenes were obtained and functional annotation was carried out using BLASTx program (http://www.ncbi.nlm.nih.gov/BLAST/) with an *E*-value threshold of 1 × 10^−5^. Public databases including the NCBI non-redundant protein database (Nr) (http://www.ncbi.nlm.nih.gov), the SwissProt database (http://www.expasy.ch/sprot), the KEGG (http://www.genome.jp/kegg), and the COG/KOG database (http://www.ncbi.nlm.nih.gov/COG) were used to annotate the *M. canadensis* unigenes. TFs were identified by aligning unigenes to the PlantTFDB database [52].

### 4.4. Differentially Expressed Genes Analysis

The abundances of unigenes were calculated and normalized to RPKM [53]. To identify DEGs between MeJA-treated samples and controls, the edgeR package (http://www.r-project.org/) was used. Genes with a fold change ≥2 and a FDR <0.05 in a comparison were identified as significant DEGs. Pathway enrichment analysis was conducted by comparing gene numbers of each pathway in DEGs to genome background. The significance test of enriched pathways was determined by calculating the *p*-value and FDR correction. Heatmaps of DEGs were generated using HemI [54].

### 4.5. Quantitative Real-Time PCR

qRT-PCR was conducted to verify the expression of selected genes in *M. canadensis*. Goldenstar™ RT6 cDNA Synthesis Kit (TsingKe Biotech, Nanjing, China) was used with 1 μg of total RNA to synthesize first-strand cDNA. The qRT-PCR reactions were carried out using the qTOWER2.2 Real-Time PCR Systems (Analytik, Jena, Germany). 2 × T5 Fast qPCR Mix Kit (SYBR Green I) (TsingKe Biotech, Nanjing, China) was used to prepare qRT-PCR reactions with 2 μL of diluted cDNA as a template. The reaction systems and steps were performed according to the manufacturer’s instructions. The *M. canadensis* actin gene was used as a control to normalize the relative expression levels of target genes. All results were representative of three independent experiments. Primers used for qRT-PCR were listed in Supplementary Table S4.

## 5. Conclusions

In this study, high-throughput RNA-seq was applied to characterize the transcriptomes of *M. canadensis* treated with MeJA. A total of 81,843 unigenes were obtained and 64.55% of which could be functionally annotated in at least one database. Additionally, 4430 DEGs with 2383 up-regulated and 2047 down-regulated transcripts were identified under MeJA treatment. A lot of unigenes associated with JA signal transduction were up-regulated, which suggested that application of exogenous MeJA may regulate the JA signaling pathway in *M. canadensis*. KEGG enrichment indicated that “Monoterpenoid biosynthesis” was one of the most significantly enriched pathways in metabolism. 9 orthologous genes involved in menthol biosynthesis were identified in *M. canadensis* and the response mechanism of menthol biosynthetic genes to JA signaling needs further study, especially the possible DEGs including *GPPS-s*, *iPD*, and *MFS*. 157 differentially expressed TFs belonged to 32 families were identified and the WRKY family was the largest differentially expressed family. The number of up-regulated WRKY TFs was much higher than that of down-regulated ones (19 vs. 2). The large number of up-regulated WRKY TFs in *M. canadensis* suggests that they may play important roles in JA-responsive transcriptional regulation of secondary metabolism. However, the regulation mechanism of JA signaling on development and metabolism of *M. canadensis* still requires further characterization and putative DEGs identified in this study might be important targets. This is the first reported transcriptome study of *M. canadensis* and will provide useful information for further study of this species.

## Figures and Tables

**Table 1:** Summary of Illumina HiSeq4000 sequencing data.

Samples ^a^	Raw Reads	Clean Reads	Reads Length (bp)	Clean Bases	Q20 Percentage (%) ^b^	GC Percentage (%)
CK-1	58,914,374	57,759,958 (98.04%)	150	8,536,291,577	98.69	50.79
CK-2	58,863,024	57,904,740 (98.37%)	150	8,588,205,461	98.88	50.05
CK-3	58,109,454	57,113,908 (98.29%)	150	8,470,135,931	98.83	50.69
MeJA-1	61,076,212	59,885,296 (98.05%)	150	8,855,787,485	98.69	50.55
MeJA-2	55,398,094	54,424,124 (98.24%)	150	8,072,356,234	98.82	50.46
MeJA-3	65,765,946	64,630,794 (98.27%)	150	9,590,111,719	98.82	50.39

^a^ CK and MeJA represent libraries constructed by CK and MeJA-treated samples, respectively. Numbers indicate three biological replicates; ^b^ Q20 percentage represents percentage of bases with a Phred value >20.

**Table 2:** Statistics of assembly data.

Total assembled bases	59,279,270
Unigene number	81,843
GC percentage (%)	43.92
N50 (bp)	1126
Average length (bp)	724

**Table 3:** Functional annotations of *M. canadensis* unigenes.

Annotation Database	Number of Unigenes	Percentage (%)
Nr	52,700	64.39
Swissprot	34,565	42.23
KOG	29,536	36.09
KEGG	19,013	23.23
Annotated in at least one database	52,826	64.55
Total unigenes	81,843	100

Nr: National Center for Biotechnology Information (NCBI) non-redundant protein database; KOG: eukaryotic Orthologous Group; KEGG: Kyoto Encyclopedia of Genes and Genomes.

## Supplementary Materials

Supplementary materials can be found at http://www.mdpi.com/1422-0067/19/8/2364/s1.

## Abbreviations

MeJA	Methyl Jasmonate
CK	Control Check
MEP pathway	Methylerythritol Phosphate Pathway
GO	Gene Ontology
KOG	Eukaryotic Orthologous Group
KEGG	Kyoto Encyclopedia of Genes and Genomes
DEGs	Differentially Expressed Genes
RPKM	Reads per Kb per Million Reads
FDR	False Discovery Rate
qRT-PCR	Quantitative Real-Time PCR
TF	Transcription Factor
