# Supplementary material for: Transcriptome Analysis of JA Signal Transduction, Transcription Factors, and Monoterpene Biosynthesis Pathway in Response to Methyl Jasmonate Elicitation in Mentha canadensis L
Source: Int J Mol Sci. 2018 Aug 10;19(8):2364. doi: 10.3390/ijms19082364 (PMC6121529; doi:10.3390/ijms19082364)
Supplement: Supplementary file 1 [file ijms-19-02364-s001.zip › SupplementaryFiles/Supplementary_Figure_4.pdf]

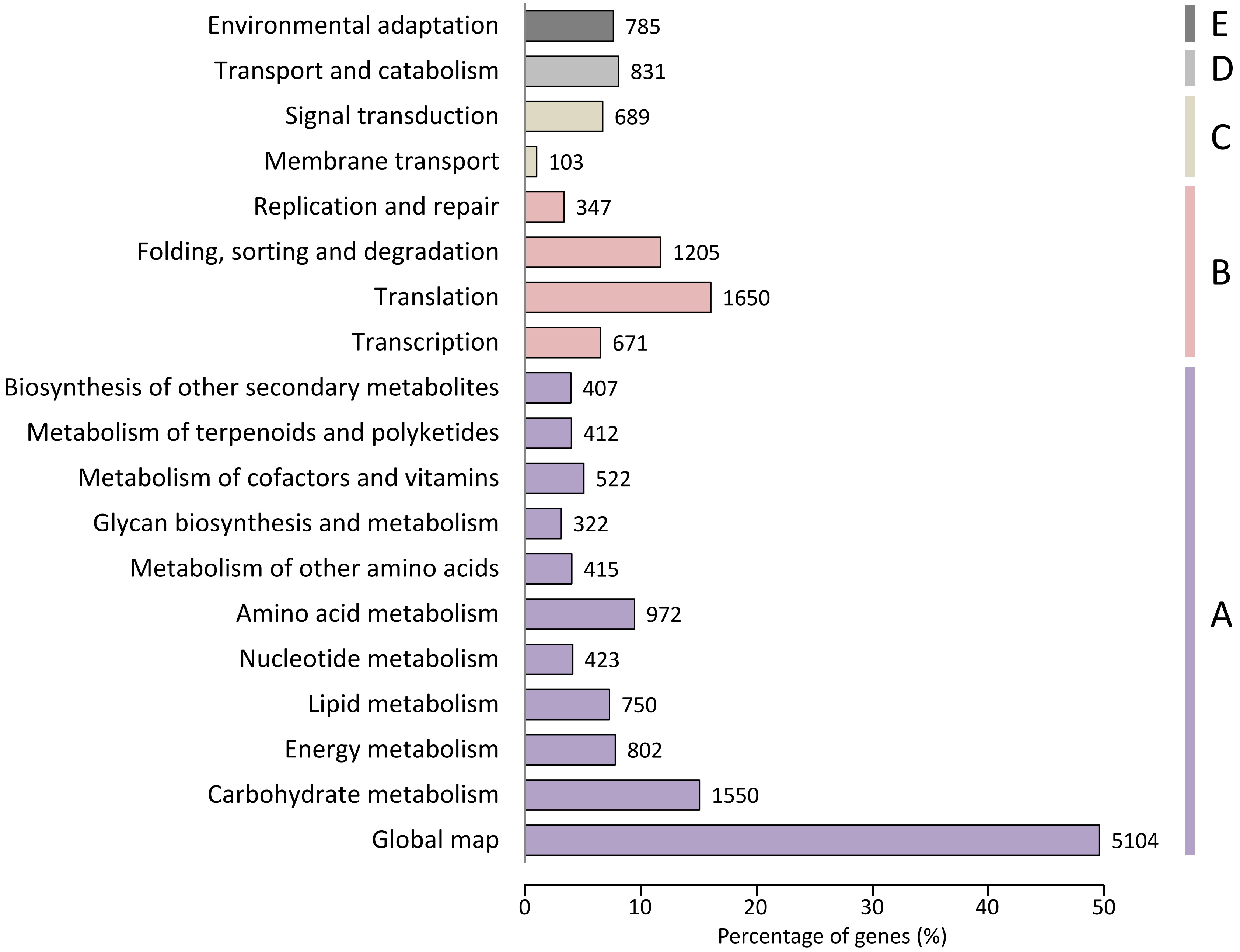

Supplementary Figure 4 KEGG pathway annotation of assembled unigenes. A, Metabolism; B, Genetic Information Processing; C, Environmental Information Processing; D, Cellular Processes; E, Organismal Systems.
