# Supplementary material for: Transcriptome Analysis of JA Signal Transduction, Transcription Factors, and Monoterpene Biosynthesis Pathway in Response to Methyl Jasmonate Elicitation in Mentha canadensis L
Source: Int J Mol Sci. 2018 Aug 10;19(8):2364. doi: 10.3390/ijms19082364 (PMC6121529; doi:10.3390/ijms19082364)
Supplement: Supplementary file 1 [file ijms-19-02364-s001.zip › SupplementaryFiles/Supplementary_Figure_6.pdf]

GPPS-l nucleotide sequences alignment

```
MpGPPS-l        ATGAGTGCTCTTGTTAATCCTGTGGCGAAATGGCCTCAGACGATCGGCGTTAAAGATGTTCACGGCGGCCGGAGGCGGAGATCCAGATCC     90
Unigene0045757  ATGAGTGTTCTTGTTAATCCTGTGACGAAATGGCCTCAGACCATCGGCGTTAGAGATGTTCACGGCGGCCGGAGGCGGAGATCCAGATCC     90

MpGPPS-l        ACTCTCTTTCAATCCCATCCACTTCGCACTGAAATGCCTTTCTCTCTCTACTTCTCATCCCCCCTCAAAGCTCCCGCCACTTTTTCCGTT    180
Unigene0045757  ACTGCCTTTCATTCTCATCCACTCCGCGCCAAACTGCCTTTCTCTCTCTACTTCTCAAGCCCTATCAAAGGTTCTGCCAGTTTTTCCGTT    180

MpGPPS-l        TCTGCAGTTTATACCAAAGAGGGCAGCGAAATTAGGGATAAAGATCCGGCGCCTTCGACTTCGCCGGCGTTCGATTTCGACGGATACATG    270
Unigene0045757  TCTGCAGTTTATACCAAAGAGGACAGCGAAATTAGGGCTGAAGATCCGGCGTCTTCGGCTTCGCCGGCGTTCGATTTCGACGGGTACATG    270

MpGPPS-l        CTCCGGAAGGCGAAATCCGTCAACAAGGCGTTGGAAGCGGCGGTGCAGATGAAGGAGCCGCTGAAGATCCACGAGTCCATGCGGTACTCC    360
Unigene0045757  CTCCGGAAGGCGAAATCCGTGAACAAGGCGTTGGAAGCGGCGGTGGAGATGAAGGAGCCGCTGAAGATCCACGAGTCCATGCGGTACTCC    360

MpGPPS-l        CTTCTCGCCGGCGGCAAGAGAGTGCGTCCTATGCTGTGCATCGCGGCCTGCGAGCTCGTCGGCGGCGACGAGTCCACGGCGATGCCGGCG    450
Unigene0045757  CTCCTCGCCGGCGGCAAGAGAGTGCGTCCGATTCTGTGCATCGCCGCCTGCGAGCTCGTCGGCGGCGACGAGACCACGGCGATGCCGGCG    450

MpGPPS-l        GCCTGCGCCGTCGAGATGATCCACACGATGTCGCTGATGCACGACGACCTCCCATGCATGGACAACGACGACCTCCGCCGCGGCAAGCCG    540
Unigene0045757  GCCTGCGCCGTCGAGATGATCCACACGATGTCGCTGATGCACGACGACCTCCCCTGCATGGACAACGACGACCTCCGCCGCGGCAAGCCG    540

MpGPPS-l        ACGAACCACATGGCTTTCGGCGAGAGCGTGGCGGTCCTCGCCGGCGACGCCCTCCTCTCCTTCGCGTTCGAGCACGTGGCGGCGGCGACC    630
Unigene0045757  ACGAACCACAAGGTATTCGGCGAGAGCACGGCGGTCCTCGCCGGCGACGCACTCCTCTCCTTCGCGTTCGAGCACGTGGCGGCGGCGACG    630

MpGPPS-l        AAAGGCGCGCCGCCGGAGCGGATCGTGAGGGTCCTCGGCGAGCTGGCTGTCTCGATCGGGTCGGAGGGGCTGGTGGCGGGGCAGGTGGTG    720
Unigene0045757  AGGGGCGCGCCGACGGAGCGGATCGTGAGGGTCCTCGGCGAGCTGGCGGTGTCGATCGGGTCGGAGGGGCTGGTGGCGGGGCAGGTTGTG    720

MpGPPS-l        GACGTCTGCTCGGAGGGGATGGCGGAGGTCGGGCTGGACCACCTCGAGTTCATCCACCACCACAAGACGGCGGCGCTGCTGCAGGGGTCG    810
Unigene0045757  GACATCTGCTCGGAGGGGATGGCGGAGGTCGGGTTGGAGCACCTCGAGTACATCCACCACCACAAGACGGCGGCGCTGCTGCAGGGTTCG    810

MpGPPS-l        GTGGTTCTGGGGGCGATTTTGGGCGGCGGAAAGGAGGAGGAGGTGGCGAAGCTGAGAAAATTCGCGAATTGCATCGGATTGCTGTTTCAG    900
Unigene0045757  GTGGTTTTGGGGGCGATTTTGGGCGGCGGAGGCGAGGAGGAGGTGGCGAGGCTCAGGAAATTCGCGAATTGCATCGGATTGCTGTTTCAG    900

MpGPPS-l        GTGGTGGACGATATCCTAGATGTGACGAAATCGTCCAAGGAATTGGGGAAGACGGCGGGGAAGGATCTGGTGGCGGATAAAACCACATAT    990
Unigene0045757  GTTGTGGACGACATCCTCGATGTGACGAAATCGTCAAAGGAATTGGGGAAGACGGCGGGGAAGGATCTGGTGGCGGACAAAACGACATAT    990

MpGPPS-l        CCGAAGCTAATAGGCGTGGAGAAATCCAAGGAATTCGCGGATCGGTTGAACAGGGAGGCGCAGGAGCAGCTCCTCCATTTTCATCCTCAT   1080
Unigene0045757  CCGAAGCTGATAGGAGTGGAGAAATCGAAGGAATTCGCAGATCGGCTGAAGAGGGAGGCGGTGGAGCAGCTCCTCCATTTCCATCCTCAC   1080

MpGPPS-l        AGGGCAGCTCCATTGATTGCTCTCGCCAATTATATTGCTTATAGGGACAATTG                                      1133
Unigene0045757  AGGGCAGCTCCATTGATTGCTCTCGCCAATTACATTGCTTACAGAGACAATTG                                      1133
```

GPPS-l nucleotide sequences alignment

```
MpGPPS-l        MSALVNPVAKWPQTIGVKDVHGGRRRRSRSTLFQSHPLRTEMPFSLYFSSPLKAPATFSVSAVYTKEGSEIRDKDPAPSTSPAFDFDGYM     90
Unigene0045757  MSVLVNPVTKWPQTIGVRDVHGGRRRRSRSTAFHSHPLRAKLPFSLYFSSPIKGSASFSVSAVYTKEDSEIRAEDPASSASPAFDFDGYM     90

MpGPPS-l        LRKAKSVNKALEAAVQMKEPLKIHESMRYSLLAGGKRVRPMLCIAACELVGGDESTAMPAACAVEMIHTMSLMHDDLPCMDNDDLRRGKP    180
Unigene0045757  LRKAKSVNKALEAAVEMKEPLKIHESMRYSLLAGGKRVRPILCIAACELVGGDETTAMPAACAVEMIHTMSLMHDDLPCMDNDDLRRGKP    180

MpGPPS-l        TNHMAFGESVAVLAGDALLSFAFEHVAAATKGAPPERIVRVLGELAVSIGSEGLVAGQVVDVCSEGMAEVGLDHLEFIHHHKTAALLQGS    270
Unigene0045757  TNHKVFGESTAVLAGDALLSFAFEHVAAATRGAPTERIVRVLGELAVSIGSEGLVAGQVVDICSEGMAEVGLEHLEYIHHHKTAALLQGS    270

MpGPPS-l        VVLGAILGGGKEEEVAKLRKFANCIGLLFQVVDDILDVTKSSKELGKTAGKDLVADKTTYPKLIGVEKSKEFADRLNREAQEQLLHFHPH    360
Unigene0045757  VVLGAILGGGGEEEVARLRKFANCIGLLFQVVDDILDVTKSSKELGKTAGKDLVADKTTYPKLIGVEKSKEFADRLKREAVEQLLHFHPH    360

MpGPPS-l        RAAPLIALANYIAYRD                                                                           376
Unigene0045757  RAAPLIALANYIAYRD                                                                           376
```

GPPS-s nucleotide sequences alignment

```
MpGPPS-s        ATGGCCATTAATCTCTCCCATATCAACTCCAAAACATGTTTCCCTCTCAAAACAAGATCTGATCTCAGCCGTTCTTCTTCCGCGCGTTGC   90
Unigene0038587  ATGGCCATTAATCTCTCCCATATCAACTCCAAAACATGTTTCCCTCTCAAAACAAGATCTGATCTCAGCCGTTCTTCTTCCGTGCGTTGC   90

MpGPPS-s        ATGCCAACTGCCGCCGCTGCCGCCTTCCCCACTATCGCCACCGCCGCCCAAAGTCAGCCGTACTGGGCCGCCATCGAGGCCGACATAGAG  180
Unigene0038587  ATGCCAACTGCCGCCGCTGCCGTCTTCCCCACTATCGCCACTGCCGCCCAAAGTCAGCCGTATTGGGCCGCCATCGGGGCCGACATCGAC  180

MpGPPS-s        AGATACCTGAAGAAATCCATCACAATAAGGCCGCCGGAGACAGTTTTCGGGCCCATGCACCACCTCACCTTCGCCGCCCCAGCCACCGCC  270
Unigene0038587  AGATACCTGAAGAAATCCATCCCAATAAGGCCGCCGGAGACTGTTTTCGGGCCCATGCACCACCTCACCTTCGCCGCCCCAGCCACCGCC  270

MpGPPS-s        GCCTCCACCCTATGCTTGGCGGCGTGCGAGCTCGTCGGCGGCGACCGAAGCCAAGCCATGGCAGCCGCGGCGGCGATCCATCTCGTGCAC  360
Unigene0038587  GCCTCCGCCCTATGCTTGGCGGCGTGCGAGCTCGTCGGCGGCGACCGAAGCCAAGCCATGGCAGCCGCGGCGGCGATCCATCTCGTGCAC  360

MpGPPS-s        GCGGCAGCCTACGTCCACGAGCACCTCCCTCTAACCGACGGGTCGAGGCCCGTATCCAAGCCCGCAATCCAGCACAAGTACGGCCCGAAC  450
Unigene0038587  GCGGCAGCCTACGTCCACGAACACCTCCCTCTAACCGACGGGTCGAGGCCCGAATCCAAGCCCGCAATCCAGCACAAGTACGGCCCGAAC  450

MpGPPS-s        GTCGAGCTCCTCACCGGAGACGGGATTGTCCCGTTCGGGTTTGAGTTGCTGGCCGGGTCAGTGGACCCGGCCCGAACAGACGACCCGGAT  540
Unigene0038587  GTCGAGCTCCTCACCGGAGACGGGATTGTCCCGTTCGGGTTTGAGTTGCTGGCCGGGTCAGTGGACCCGGCCCGAACAGACGACCCGGAT  540

MpGPPS-s        AGGATTCTGAGAGTTATAATAGAGATCAGTCGGGCCGGCGGGCCGGAGGGAATGATAAGCGGGCTGCATAGGGAAGAAGAAATTGTTGAT  630
Unigene0038587  AGGATCCTGAGAGTTATAATAGAGATCAGTCGGGCCGGCGGGCCGGAGGGAATGATAAGCGGGCTGCATAGGGAAGAAGAAATTGTTGAT  630

MpGPPS-s        GGAAATACGAGTTTAGACTTCATTGAATATGTGTGCAAGAAAAAATACGGCGAGATGCATGCTTGCGGCGCGGCTTGTGGAGCCATATTG  720
Unigene0038587  GGAAATACGAGTTTAGACTTCATTGAATATGTGTGCAAGAAAAAATACGGCGAGATGCATGCTTGCGGCGCGGCTTGTGGAGCCATATTG  720

MpGPPS-s        GGCGGCGCAGCCGAGGAGGAGATTCAGAAGCTGAGGAATTTCGGGCTTTATCAAGGAACTCTCAGAGGAATGATGGAAATGAAAAATTCT  810
Unigene0038587  GGCGGCGCAACCGATGAGGAGATTCAGAAGCTGAGGAATTTCGGGCTTTATCAAGGAACTCTCAGAGGAATGATGGAAATGAAAAATTCT  810

MpGPPS-s        CATCAATTAATTGATGAGAATATAATTGGAAAATTGAAAGAATTGGCTCTCGAGGAGTTGGGAGGCTTCCACGGGAAGAACGCTGAGCTG  900
Unigene0038587  CATGAATTAATTGATGAGAATATAATTGGAAAATTGAAAGAATTGGCTCTCGAGGAGTTGGGAGGCTTCCACGGGAAGAACGCTGAGCTG  900

MpGPPS-s        ATGTCGAGCCTTGTAGCCGAGCCGAGCCTTTACGCGGCTTA                                                    941
Unigene0038587  ATGTCGAGCCTTGTAGCCGAGCCGAGCCTTTGCGCGGCTTA                                                    941
```

GPPS-s amino acid sequences alignment

```
MpGPPS-s        MAINLSHINSKTCFPLKTRSDLSRSSSARCMPTAAAAAFPTIATAAQSQPYWAAIEADIERYLKKSITIRPPETVFGPMHHLTFAAPATA   90
Unigene0038587  MAINLSHINSKTCFPLKTRSDLSRSSSVRCMPTAAAAVFPTIATAAQSQPYWAAIGADIDRYLKKSIPIRPPETVFGPMHHLTFAAPATA   90

MpGPPS-s        ASTLCLAACELVGGDRSQAMAAAAAIHLVHAAAYVHEHLPLTDGSRPVSKPAIQHKYGPNVELLTGDGIVPFGFELLAGSVDPARTDDPD  180
Unigene0038587  ASALCLAACELVGGDRSQAMAAAAAIHLVHAAAYVHEHLPLTDGSRPESKPAIQHKYGPNVELLTGDGIVPFGFELLAGSVDPARTDDPD  180

MpGPPS-s        RILRVIIEISRAGGPEGMISGLHREEEIVDGNTSLDFIEYVCKKKYGEMHACGAACGAILGGAAEEEIQKLRNFGLYQGTLRGMMEMKNS  270
Unigene0038587  RILRVIIEISRAGGPEGMISGLHREEEIVDGNTSLDFIEYVCKKKYGEMHACGAACGAILGGATDEEIQKLRNFGLYQGTLRGMMEMKNS  270

MpGPPS-s        HQLIDENIIGKLKELALEELGGFHGKNAELMSSLVAEPSLYA                                                   312
Unigene0038587  HELIDENIIGKLKELALEELGGFHGKNAELMSSLVAEPSLCA                                                   312
```

LS nucleotide sequences alignment

```
MpLS            ATGGCTCTCAAAGTGTTTAGTGGTGCAATGCAAATGCCAATTCCTAGCAAGCTAACGACCTATCTTCAACCCTCACACTTGAATTCTTCT      90
Unigene0018537  ATGGCTCTCACAGTGTTTAGTGGTGCAATGCAAATGGCAATTCCTAGCACGCTAACGACCTATCTTCAACCCTCACACTTGAATTCTTCT      90

MpLS            CCTAAATTGCTCTCTAACACTAAGGGTACTAGTCGATCTCGCCTCCGTGTGTCTTGCTCCTCCTCGCAACTCACTACCGAAAGACGATCC     180
Unigene0018537  CCTAAATTGCTCTCTAACACTAAGGGTACTAGTCGGTCTCGCCTCCGTGTGTCTTGCTCCTCCTCGCAACTCACTACCGAAAGACGATCC     180

MpLS            GGAAACTACAACCCTTCTCGTTGGGATGTCGACTTTATCCAAACGCTCCACAGTGATTATAAGGACGAGAAGCATGCGAGAAGGGCTTCT     270
Unigene0018537  GGAAACTACAACCCTTCTCGTTGGGATGTCGACTTTATCCAAACGCTCCATAGTGATTATAAGGACGAGAAGCATGCGAGAAGGGCTTCT     270

MpLS            GAGCTGGTCACTTTGGTGAAGATGGAATTGGAGAAAGAAACGGATCAAATTCGACAGCTTGAATTGATTGATGACTTGCAGAGGATGGGG     360
Unigene0018537  GAGCTGGTCACTTTGGTGAAGATGGAATTGGAGAAAGAAACGGATCAAATTCGACAGCTTGAATTGATTGATGACTTGCAGAGGATGGGG     360

MpLS            CTGTCCGATCATTTCCAGAACGAGTTCAAAGAAATCTTGTCCTCTGTATATCTCGACCATGGCTATTACAAGAATCCTGATCCAAAAGAA     450
Unigene0018537  CTGTCCGATCATTTCCAGAACGAGTTCAAAGAAATCTTGTCCTCTGTATATCTCGACCATGACTATTACAAGAATCCTGATCCAAAAGAA     450

MpLS            GAAAGGGATCTATACTCCACATCTCTTGCATTTAGGCTCCTTCGAGAACATGGGTTTCAAGTTGCTCAAGAGGTATTCGACAGTTTCAAG     540
Unigene0018537  GAAAGGGATCTATACTCCACATCTCTTGCATTTAGGCTCCTTCGAGAACATGGGTTTCAAGTTGCTCAAGAGGTATTTTACGGTTTCAAG     540

MpLS            AACGAGGAGGGTGAGTTCAAAGAAAGCCTTAGCGACGACACCAGAGGATTGTTGCAACTGTACGAAGCTTCTTTTCTGTTGACGGAAGGC     630
Unigene0018537  AACGA...GGGTGAGTTCAAAGAAAGCCTTAGCGACGACACCAGAGGATTGTTGGAACTGTACGAAGCTTCTTTTCTGTTGACGGAAGGC     627

MpLS            GAAACGACGCTCGAGTCAGCGAGAGAATTCGCCACAAAATTTCTGGAAGAAAGAGTGAATGAGGGTGGTGGTGATGAAAACCTTTTAACA     720
Unigene0018537  GAAACGACGCTCGAGTCAGCGAGAGAATTCGCCACAAAATTTCTGGAAGAAAGAGTGAATGAGGGTGGTGGTGATGAAAACCTTTTAACA     717

MpLS            AGAATCGCATATTCTTTAGAAATCCCACTTCATTGGAGGATTAAAAGGCCAAATGCACCTGTGTGGATCGATTCGTATAGGAAGAGGCCC     810
Unigene0018537  AGAATCGCATATTCTTTAGAAATCCCACTTCATTGGAGGATTAAAAGGCCAAATGCACCTGTGTGGATTGATTCGTATAGGAAGAGGCCC     807

MpLS            AACATGAATCCAGTAGTGTTGGACCTTGCCATACTCGACTTAAATATTGTTCAAGCTCATTTTCAACAAGAACTCAAAGAATCCTTCCGG     900
Unigene0018537  AACATGAATCCAGTAGTGTTGGAGCTTGCCATACTCGACTTAAATATTGTTCAAGCTCATTTTCAAGAAGAGCTCAAAGAATCCTTCAGG     897

MpLS            TGGTGGAGAAATACTGGGTTTGTTGAGAAGCTGCCCTTCGCAAGGGATAGACTGGTGGAATGCTACTTTTGGAATACTGGGATCATCGAG     990
Unigene0018537  TGGTGGAGAAATACTGGGTTTGTTGAGAAGCTGCCCTTCGCAAGGGACAGACTGGTGGAATGCTACTTTTGGAATACTGGGATCATCGAG     987

MpLS            CCACGTCAGCATGCAAGTGCAAGGATAATGATGGGCAAAGTCAACGCTCTGATTACGGTGATAGATGATATTTATGATGTCTATGGTACC    1080
Unigene0018537  CCACGTCAGCATGCAAGTGCAAGGATAATGATGGGCAAAGTCAACGCTCTGATTACGGTGATAGATGATATTTATGATGTCTATGGTACC    1077

MpLS            TTAGAAGAACTCGAACACTTCACAGACCTCATTCGGAGATGGGATATAGACTCAATCGACCAACTTCCCGATTACATGCAACTGTGCTTT    1170
Unigene0018537  TTAGAAGAACTCGAACAGTTCACAGACCTCATTCGGAGATGGGATATAGACTCAATCGACCAACTTCCCGATTACATGCAACTGTGCTTT    1167

MpLS            CTCGCACTCAACAACTTCGTCGATGAAACATCCTACGATGTTATGAAAGAGAAAGGCGTCAACGTTATACCCTATCTTCGACAATCGTGG    1260
Unigene0018537  CTTGCACTCAACAACTTCGTCGATGATACATCGTACGATGTTATGAAGGAGAAAGGCGTCAACGTTATACCCTACCTGCGGCAATCGTGG    1257

MpLS            GTGGATTTGGCGGATAAGTACATGGTAGAGGCACGGTGGTTCTACGGCGGCCACAAACCAAGTTTGGAAGAGTATTTGGAAAACTCATGG    1350
Unigene0018537  GTGGATTTGGCGGATAAGTATATGGTAGAGGCACGGTGGTTCTACGGCGGGCACAAACCAAGTTTGGAAGAGTATTTGGAGAACTCATGG    1347

MpLS            ATGTCGATAAGTGGGCCCTGCATGTTGACACACATATTTTTCCGAGTAACGGATTCATTCACAAAGGAGACTGTCGACAGTTTGTACAAG    1440
Unigene0018537  CAGTCGATAAGTGGGCCCTGTATGTTAACGCACATATTCTTCCGAGTAACAGATTCGTTCACAAAGGAGACCGTCGACAGTTTGTACAAA    1437

MpLS            TACCACGATTTAGTTCGTTGGTCATCCTTCGTTTTGCGACTTGCTGATTGATTTGGGAACCTCAGTGGAAGAGGTGAGCAGAGGCGACGTG    1530
Unigene0018537  TACCACGATTTAGTTCGCTGGTCATCCTTCGTTCTGCGGCTTGCTGACGATCTGGGAACCTCGGTGGAAGAGGTGAGCAGAGGCGATGTG    1527

MpLS            CCGAAATCACTTCAGTGCTACATGAGTGACTACAATGCGTCGGAGGCGGAGGCGCGAAAGCACGTGAAATGGCTGATAGCAGAGGTGTGG    1620
Unigene0018537  CCGAAATCACTTCAGTGCTACATGAGTGACTACAATGCATCGGAGGCGGAGGCGCGGAAGCACGTGAAATGGCTGATAGCGGAGGTGTGG    1617

MpLS            AAGAAGATGAATGCAGAGAGGGTGTCGAAGGATTCTCCATTTGGCAAAGATTTTATAGGATGTGCAGTTGATTTGGGAAGGATGGCGCAG    1710
Unigene0018537  AAGAAGATGAATGCGGAGAGGGTGTCGAAGGATTCTCCATTCGGCAAAGATTTTATAGGATGTGCAGCTGATTTAGGAAGGATGGCGCAG    1707

MpLS            TTGATGTACCATAATGGAGATGGGCATGGCACACAACATCCTATAATACATCAACAAATGACCGCAACCTTATTCGAGCCCTTTGCATG     1799
Unigene0018537  TTGATGTACCATAATGGAGATGGGCACGGCACACAACATCCTATAATACATCAACAAATGACCAGAACCTTATTCGAGCCCTTTGCATG     1796
```

LS amino acid sequences alignment

```
MpLS            MALKVFSGAMQMPIPSKLTTYLQPSHLNSSPKLLSNTKGTSRSRLRVSCSSSQLTTERRSGNYNPSRWDVDFIQTLHSDYKDEKHARRAS      90
Unigene0018537  MALTVFSGAMQMAIPSTLTTYLQPSHLNSSPKLLSNTKGTSRSRLRVSCSSSQLTTERRSGNYNPSRWDVDFIQTLHSDYKDEKHARRAS      90

MpLS            ELVTLVKMELEKETDQIRQLELIDDLQRMGLSDHFQNEFKEILSSVYLDHGYYKNPDPKEERDLYSTSLAFRLLREHGFQVAQEVFDSFK     180
Unigene0018537  ELVTLVKMELEKETDQIRQLELIDDLQRMGLSDHFQNEFKEILSSVYLDHDYYKNPDPKEERDLYSTSLAFRLLREHGFQVAQEVFYGFK     180

MpLS            NEEGEFKESLSDDTRGLLQLYEASFLLTEGETTLESAREFATKFLEERVNEGGGDENLLTRIAYSLEIPLHWRIKRPNAPVWIDSYRKRP     270
Unigene0018537  NE.GEFKESLSDDTRGLLELYEASFLLTEGETTLESAREFATKFLEERVNEGGGDENLLTRIAYSLEIPLHWRIKRPNAPVWIDSYRKRP     269

MpLS            NMNPVVLDLAILDLNIVQAHFQQELKESFRWWRNTGFVEKLPFARDRLVECYFWNTGIIEPRQHASARIMMGKVNALITVIDDIYDVYGT     360
Unigene0018537  NMNPVVLELAILDLNIVQAHFQEELKESFRWWRNTGFVEKLPFARDRLVECYFWNTGIIEPRQHASARIMMGKVNALITVIDDIYDVYGT     359

MpLS            LEELEHFTDLIRRWDIDSIDQLPDYMQLCFLALNNFVDETSYDVMKEKGVNVIPYLRQSWVDLADKYMVEARWFYGGHKPSLEEYLENSW     450
Unigene0018537  LEELEQFTDLIRRWDIDSIDQLPDYMQLCFLALNNFVDDTSYDVMKEKGVNVIPYLRQSWVDLADKYMVEARWFYGGHKPSLEEYLENSW     449

MpLS            MSISGPCMLTHIFFRVTDSFTKETVDSLYKYHDLVRWSSFVLRLADDLGTSVEEVSRGDVPKSLQCYMSDYNASEAEARKHVKWLIAEVW     540
Unigene0018537  QSISGPCMLTHIFFRVTDSFTKETVDSLYKYHDLVRWSSFVLRLADDLGTSVEEVSRGDVPKSLQCYMSDYNASEAEARKHVKWLIAEVW     539

MpLS            KKMNAERVSKDSPFGKDFIGCAVDLGRMAQLMYHNGDGHGTQHPIIHQQMTATLFEPF                                     598
Unigene0018537  KKMNAERVSKDSPFGKDFIGCAADLGRMAQLMYHNGDGHGTQHPIIHQQMTRTLFEPF                                     597
```

L3OH nucleotide sequences alignment

```
MpL3OH-PM2       ATGGAGCTCCTCCAGCTTTGGTCGGCGCTTATAATCCTCGTAGTAACCTACACCATATCCCTCCTAATCAACCAATGGCGAAAACCGAAA    90
Unigene0052571   ATGGAGCTCCTCCAGCTTTGGTCGGCGCTTATAATCCTCGTCGTAACCTACGCCATATCTCTCCTAATCAACCAATGGCGAAAACCGAAA    90

MpL3OH-PM2       CCCCAAGGGAAGTTCCCCCCGGGCCCGCCGAAGCTGCCGCTGATCGGGCACCTCCACCTCCTGTGGGGGAAGCTGCCGCAGCACGCGCTG   180
Unigene0052571   CCCCAAGAGAAGTTCCCCCCGGGCCCGCCGAAGCTGCCGCTGATCGGGCACCTCCACCTCCTGTGGGGGAAGCTGCCGCAGCACGCGCTG   180

MpL3OH-PM2       GCCAGCGTGGCGAAGGAGTACGGCCCCGTGGCCCACGTGCAGCTGGGTGAGGTGTTCTCCGTCGTCCTTTCGTCGCGGGAGGCGACGAAG   270
Unigene0052571   GCCAGCGTGGCAAAGGAGTACGGCCCCGTGGCCCACGTGCAGCTGGGTGAGGTGTTCTCCGTCGTCCTTTCGTCGCGGGAGGCGACGAAG   270

MpL3OH-PM2       GAGGCGATGAAGCTGGTAGACCCGGCGTGCGCGAACCGGTTCGAGAGCATCGGGACGAGGATCATGTGGTACGACAACGAGGACATCATC   360
Unigene0052571   GAGGCGATGAAGCTGGTGGACCCGGCGTGCGCGAACCGGTTCGAGAGCATCGGGACAAGGATCATGTGGTACGACAACGAGGACATCATC   360

MpL3OH-PM2       TTCAGCCCCTACAGCGAGCACTGGCGCCAGATGCGCAAGATCTGCGTCTCCGAGCTCCTCTCCTCCCGCAACGTCCGCTCCTTCGGCTTC   450
Unigene0052571   TTCAGCCCCTACAGCGAGCACTGGCGCCAGATGCGCAAGATCTGCGTCTCCGAGCTCCTCTCCTCCCGCAACGTCCGCTCCTTCGGCTTC   450

MpL3OH-PM2       ATCCGGCAGGACGAGGTGTCGCGCCTCCTCCGCCACCTCCGCTCCTCGGCAGGGGCGGCCGTGGACATGACGGAGAGGATAGAGACGCTG   540
Unigene0052571   ATCCGGCAGGACGAGGTCTCGCGCCTCCTCCGCCACCTCCGCTCCTCGGCAGGGGCGGCCGTGGACATGACGGAGAGGATAGAGACGCTA   540

MpL3OH-PM2       ACGTGCTCCATCATCTGCAGGGCGGCGTTCGGGAGCGTGATCAGGGACAACGCGGAGCTGGTGGGGCTGGTCAAGGACGCGCTCAGCATG   630
Unigene0052571   ACGTGCTCCATCATCTGCAGGGCGGCGTTCGGGAGCGTGATCAGGGACAACGCGGAGCTGGTGGGGCTGGTCAAGGACGCGCTCAGCATG   630

MpL3OH-PM2       GCCTCGGGGTTCGAGCTCGCCGACATGTTCCCCTCCTCCAAGCTCCTCAACCTCCTCTGCTGGAACAAGAGCAAGCTCTGGAGGATGCGC   720
Unigene0052571   GCCTCGGGGTTCGAGCTCGCCGACATGTTCCCCTCCTCCAAGCTCCTCAACCTCCTCTGCTGGAACAAGAGCAAGCTCTGGAGGATGCGC   720

MpL3OH-PM2       CGCCGCGTCGACACCATCCTCGAGGCCATCGTCGACGAGCACAAGTTCAAGAAGAGCGGCGAGTTCGGCGGCGAGGACATCATCGACGTC   810
Unigene0052571   GGCCGCGTCGACACCATCCTCGAGGCCATCGTCGACGAGCACAAGCTCAAGAAGAGCGGCGAGTTCGGCGGCGAGGACATCATCGACGTC   810

MpL3OH-PM2       CTCTTCAGGATGCAGAAGGACACCCAGATCAAAGTCCCCATCACCACCAACTCCATCAAAGCCTTCATCTTCGATACGTTCTCAGCAGGG   900
Unigene0052571   CTCTTCAGGATGCAGAAGGACATCCAGATCAAAGTCCCCATCACCACCAACTCCATCAAAGCCTTCATCTTCGACACATTCTCAGCAGGG   900

MpL3OH-PM2       ACTGAGACATCCTCAACCACCACCCTATGGGTGCTGGCGGAGCTGATGAGGAACCCGGCAGTGATGGCGAAAGCGCAGGCGGAGGTGAGA   990
Unigene0052571   ACTGAGACATCCTCAACCACCACCCTATGGGTGCTGGCGGAGCTGATGAGGAACCCGGCAGTGATGGCGAAAGTGCAGGCGGAGGTGAGA   990

MpL3OH-PM2       GCGGCACTGAAGGAGAAGACGAACTGGGACGTGGATGACGTGCAAGAGCTTAAGTACATGAAATCGGTGGTGAAGGAGACGATGAGGATG  1080
Unigene0052571   GCGGCACTGAAGGGGAAGACGAATTGGGACGTGGACGACGTGCAAGAGCTTAAGTACATGAAATCGGTGGTGAAGGAGACGATGAGGATG  1080

MpL3OH-PM2       CACCCTCCGATCCCGTTGATCCCGAGATCATGCAGAGAAGAATGCGTGGTTAACGGGTATACGATTCCGAACAAGGCCAGAATCATGATC  1170
Unigene0052571   CACCCTCCGATCCCGTTGATCCCGAGATCATGCAGAGAAGAATGCGTGGTTAACGGGTATACGATTCCGAACAAGGCCAGAATCATGATC  1170

MpL3OH-PM2       AACGTCTGGTCCATGGGCAGGAATCCTCTCTACTGGGAAAAACCCGATACCTTTTGGCCCGAAAGGTTTGACCAAGTTTCAAAGGATTTC  1260
Unigene0052571   AACGTCTGGTCCATGGGCAGGAATCCTCTCTACTGGGAAAAACCCGAGACCTTTTGGCCCGAAAGGTTTGACGATGTTTCAAAGGATTTC  1260

MpL3OH-PM2       ATGGGAAATGATTTCGAGTTCGTCCCGTTCGGAGCGGGAAGAAGAATCTGCCCCGGCTTGAACTTCGGGTTGGCAAACGTTGAGGTTCCA  1350
Unigene0052571   ATGGGAAATGATTTCGAGTTCGTCCCGTTCGGAGCGGGAAGAAGAATCTGCCCCGGCTTGAACTTCGGGTTGGCAAACGTTGAGGTTCCA  1350

MpL3OH-PM2       TTGGCGCAGCTTCTTTACCACTTCGACTGGAAGTTGGCGGAAGGAATGAAACCTTCTGATATGGACATGTCTGAGGCGGAAGGCCTTACC  1440
Unigene0052571   TTGGCGCAGCTTCTTTACCACTTCGACTGGAAGTTGGCGGAAGGGATGAAACCTTCTGATATGGACATGTCTGAGGCGGAAGGCCTTACC  1440

MpL3OH-PM2       GGAATACTAAAGAACAATCTTCTTCTTGTTCCCACACCCTACGATCCTTCATCATG                                  1496
Unigene0052571   GGAATACGAAAGAACAATCTTCTTCTTGTTCCCACACCCTACGATCCTTCCTTATG                                  1496
```

L3OH amino acid sequences alignment

```
MpL3OH-PM2       MELLQLWSALIILVVTYTISLLINQWRKPKPQGKFPPGPPKLPLIGHLHLLWGKLPQHALASVAKEYGPVAHVQLGEVFSVVLSSREATK    90
Unigene0052571   MELLQLWSALIILVVTYAISLLINQWRKPKPQEKFPPGPPKLPLIGHLHLLWGKLPQHALASVAKEYGPVAHVQLGEVFSVVLSSREATK    90

MpL3OH-PM2       EAMKLVDPACANRFESIGTRIMWYDNEDIIFSPYSEHWRQMRKICVSELLSSRNVRSFGFIRQDEVSRLLRHLRSSAGAAVDMTERIETL   180
Unigene0052571   EAMKLVDPACANRFESIGTRIMWYDNEDIIFSPYSEHWRQMRKICVSELLSSRNVRSFGFIRQDEVSRLLRHLRSSAGAAVDMTERIETL   180

MpL3OH-PM2       TCSIICRAAFGSVIRDNAELVGLVKDALSMASGFELADMFPSSKLLNLLCWNKSKLWRMRRRVDTILEAIVDEHKFKKSGEFGGEDIIDV   270
Unigene0052571   TCSIICRAAFGSVIRDNAELVGLVKDALSMASGFELADMFPSSKLLNLLCWNKSKLWRMRGRVDTILEAIVDEHKLKKSGEFGGEDIIDV   270

MpL3OH-PM2       LFRMQKDTQIKVPITTNSIKAFIFDTFSAGTETSSTTTLWVLAELMRNPAVMAKAQAEVRAALKEKTNWDVDDVQELKYMKSVVKETMRM   360
Unigene0052571   LFRMQKDIQIKVPITTNSIKAFIFDTFSAGTETSSTTTLWVLAELMRNPAVMAKVQAEVRAALKGKTNWDVDDVQELKYMKSVVKETMRM   360

MpL3OH-PM2       HPPIPLIPRSCREECVVNGYTIPNKARIMINVWSMGRNPLYWEKPDTFWPERFDQVSKDFMGNDFEFVPFGAGRRICPGLNFGLANVEVP   450
Unigene0052571   HPPIPLIPRSCREECVVNGYTIPNKARIMINVWSMGRNPLYWEKPETFWPERFDDVSKDFMGNDFEFVPFGAGRRICPGLNFGLANVEVP   450

MpL3OH-PM2       LAQLLYHFDWKLAEGMKPSDMDMSEAEGLTGILKNNLLLVPTPYDPS                                           497
Unigene0052571   LAQLLYHFDWKLAEGMKPSDMDMSEAEGLTGIRKNNLLLVPTPYDPS                                           497
```

iPD nucleotide sequences alignment

```
MpiPD            ATGGCAAGCGTGAAGAAGCTCGCAGGCAAGGTAGCCATCGTAACCGGCGGCGCCAGCGGCATCGGCGAGGTCACCGCCCGCCTCTTCGCC   90
Unigene0033747   ATGGCAATCGTGAAGAAGCTCGCAGGCAAGGTAGCCATCGTAACCGGCGGCGCCAGCGGCATCGGCGAGGTCACCGCCCGCCTCTTCGCC   90

MpiPD            GAGCGCGGCGCCCGCGCAGTGGTGATCGCCGACATGCAGCCCGAGAAGGGCGGTACCGTGGCGGAATCCATAGGCGGCCGGCGGTGCAGC   180
Unigene0033747   GAGCGCGGCGCCCGCGCGGTGGTGATCGCCGACATGCAGCCCGAGAAGGGCGGTACCGTGGCGGAATCCATCGGTGGCCGGCGGTGCAGC   180

MpiPD            TACGTCCACTGCGACATCACCGACGAGCAACAGGTCAGGTCCGTCGTGGATTGGACCGCCGCCACCTACGGCGGCGTCGACGTGATGTTC   270
Unigene0033747   TACGTCCACTGCGACATCACCGACGAGGAACAGGTCAGGTCCGTCGTGGATTGGACCGCCGCCACCTACGGCGGCGTCGACGTGATGTTC   270

MpiPD            TGCAACGCCGGCACCGCCAGCGCCACCGCTCAGACCGTCCTGGACCTGGACCTGGCGCAGTTCGACCGCGTGATGCGTGTCAACGCCCGT   360
Unigene0033747   TGCAACGCGGGCACCGCCAGCGCCACCGCTCAGACCGTCCTGGACCTGGACCTGGCGCAGTTCGACCGCGTCATGCGCGTCAACGCCCGT   360

MpiPD            GGCACGGCGGCGTGCGTGAAGCAGGCGGCGCGTAAGATGGTGGAGCTGGGGAGGGGCGGCGCTATCATCTGCACCGCCAGCGCGACGGTG   450
Unigene0033747   GGCACGGCGGCGTGCGTGAAGCAGGCGGCGCGGAAGATGGTGGAGCTGGGGAGGGGAGGCGCTATCATCTGCACCGCCAGCGCGACGGCG   450

MpiPD            CACCACGCCGGGCCCAACTTGACGGACTACATCATGTCGAAGTGCGGGGTGCTGGGGCTGGTGCGGTCGGCGAGTTTGCAGCTCGGGGTG   540
Unigene0033747   AACCACGCCGGTCCCAACTTGACGGACTACATCATGTCGAAGTGCGGGGTGCTGGGGCTGGTGCGGTCGGCGAGTTTGCAGCTCGGGGTG   540

MpiPD            CACGGGATTAGGGTTAACAGCGTGTCGCCGACGGCGCTGGCCACGCCGCTCACCGCGACGATCGGGCTCCGGACGGCCGCCGATGTGGAG   630
Unigene0033747   CACGGGATTAGGGTTAACAGCGTGTCGCCGACGGCGCTGGCCACGCCGCTCACCGCGACGATCGGGCTCCGGACGGCCGCCGATGTGGAG   630

MpiPD            AGCTTCTATGGGCAGGTCACGAGCTTGAAAGGGGTGGCGATCACGGCGGAGCACGTGGCGGAGGCGGTGGCATTTCTGGCTTCGGATGAG   720
Unigene0033747   AGCTTCTATGGGCAGGTCACGAGCTTGAAAGGGGTGGCGATCACGGCGGAGCACGTGGCCGAGGCGGTGGCGTTTCTGGCTTCGGATGAG   720

MpiPD            GCGGCGTTCGTCACCGGCCATGATTTGGCGGTGGATGGTGGACTGCAGTGTTTACCATTCGTGGCCGTGGCCAAGTG             797
Unigene0033747   GCGGCGTTCGTCACCGGCCATGATTTGGCGGTGGATGGTGGACTGCAGTGTTTACCATTCGTGGCCGTGGCCAAGTG             797
```

iPD amino acid sequences alignment

```
MpiPD            MASVKKLAGKVAIVTGGASGIGEVTARLFAERGARAVVIADMQPEKGGTVAESIGGRRCSYVHCDITDEQQVRSVVDWTAATYGGVDVMF   90
Unigene0033747   MAIVKKLAGKVAIVTGGASGIGEVTARLFAERGARAVVIADMQPEKGGTVAESIGGRRCSYVHCDITDEEQVRSVVDWTAATYGGVDVMF   90

MpiPD            CNAGTASATAQTVLDLDLAQFDRVMRVNARGTAACVKQAARKMVELGRGGAIICTASATVHHAGPNLTDYIMSKCGVLGLVRSASLQLGV   180
Unigene0033747   CNAGTASATAQTVLDLDLAQFDRVMRVNARGTAACVKQAARKMVELGRGGAIICTASATANHAGPNLTDYIMSKCGVLGLVRSASLQLGV   180

MpiPD            HGIRVNSVSPTALATPLTATIGLRTAADVESFYGQVTSLKGVAITAEHVAEAVAFLASDEAAFVTGHDLAVDGGLQCLPFVAVA         264
Unigene0033747   HGIRVNSVSPTALATPLTATIGLRTAADVESFYGQVTSLKGVAITAEHVAEAVAFLASDEAAFVTGHDLAVDGGLQCLPFVAVA         264
```

iPR nucleotide sequences alignment

```
MpiPR           ATGGCAGAAGTACAGAGGTATGCATTGGTGACGGGAGCAAACAAAGGAATCGGGTTCGAAATCTGCAGGCAGTTGGCAGAAAAAGGAATT   90
Unigene0033916  ATGGCAAATGTACAGAGGTATGCATTGGTGACGGGAGCAAATAAAGGAATCGGGTTCGAAATCTGCAGGCAGTTAGCAGAAAAAGGAATC   90

MpiPR           ATAGTAATTTTAACATCGAGAAATGAAAAGAGAGGCCTCGAAGCTCGACAAAAGCTGCTCAAGGAGTTGAATGTTTCTGAAAATCGTTTA  180
Unigene0033916  ATTGTGATTTTAACGGCAAGAAATGAAAAGAGAGGCATCGAAGCTCACCAAAGGCTGCTTAAGGAATTAAACATTTCTAAAAATCATTTG  180

MpiPR           GTTTTTCATCAGCTTGATGTTACTGATCTAGCTAGCGTTGCTGCTGTTGCTGTCTTTATCAAATCTAAATTCGGAAAGCTTGATATTCTG  270
Unigene0033916  GTTTTTCATCAACTCGATGTTACCGATCCTGCTAGCATCGCCGCTGTTGCCGTCTTCATCAAATCTACATTCGGAAAACTCGATATTCTG  270

MpiPR           GTGAATAATGCAGGAGTTAGCGGAGTAGAGATGGTTGGAGATGTTTCTGTGTTCAATGAGTATATTGAGGCTGACTTCAAAGCCCTTCAA  360
Unigene0033916  GTGAACAATGCCGGAGTTAGCGGAGTAGAGATGGTTGGAGATGTTTCCGTGTTCAACGAGTATATTGAGGCTGACTTCAACGCCCTTCAA  360

MpiPR           GCACTCGAAGCTGGTGCAAAGGAAGAGCCGCCATTTAAGCCAAAAGCCAATGGAGAAATGATCGAAAAATTCGAAGGAGCCAAAGATTGC  450
Unigene0033916  GCACTCGAAGCTGGTGCAAAGGAAGAGCCGCCATTTAAGCCCAAAGCAAATGGAGAAATGATCGAAAAATTCGAAGGAGCCAAAGATTGC  450

MpiPR           GTTGTAACAAACTACTACGGTCCAAAGAGACTAACACAAGCCCTCATTCCTCTCTTACAACTATCTCCTTCACCGAGAATCGTCAACGTC  540
Unigene0033916  GTCGAAACAAACTACTATGGTCCAAAGAGACTAACACAAGCCCTCATTCCTCTCTTACAACTATCTCCTTCGCCGAGAATCGTCAACGTC  540

MpiPR           TCCTCCTCCTTCGGGAGTTTACTGCTACTGTGGAACGAATGGGCAAAGGGAGTGTTGGGCGACGAGGACAGGCTGACAGAAGAGAGAGTG  630
Unigene0033916  TCCTCCTCCTTCGGGAGTTTACTGCTTCTGTGGAACGAATGGGCAAAGGGAGTGCTGGGGGACGAGGATAGTCTGACGGAAGAGAGAGTG  630

MpiPR           GACGAAGTCGTGGAGGTTTTTCTCAAAGATATAAAAGAAGGTAAGCTTGAAGAAAGCCAATGGCCTCCACATTTTGCGGCGGAAAGAGTT  720
Unigene0033916  GACGAAGTAGTGGAGGTTTTTCTCAAGGATATAAAAGAAGGTAAGCTCGAAGAAAATCAATGGCCTCCACATTTTGCGGCGGAAAGAGTC  720

MpiPR           TCGAAGGCGGCGTTGAACGCATACACTAAGATTGCGGCGAAGAAGTACCCGAGTTTCCGCATAAATGCAATATGCCCGGGTTATGCGAAA  810
Unigene0033916  TCGAAGGCGGCGCTGAACGCGTACACTAAGATCGCGGCGAAGAAGTACCCGAGTTTCCGCATAAATGCGATATGCCCCGGTTATGCGAAA  810

MpiPR           ACCGACATTACCTTCCATGCGGGCCCACTGAGTGTGGCCGAAGCTGCTCAAGTTCCGGTGAAGCTGGCTCTGCTGCCTGATGGGGGGCCC  900
Unigene0033916  ACCGACATTACCTTCCATGCGGGACCTTTGAGTGTGTCGGAAGCTGCCCAGGTTCCGGTGAAGCTGGCTCTGCTGCCTGATGGTGGGCCC  900

MpiPR           TCCGGCTGCTTCTTCCCTCGAGACAAAGCTTTGGCTCTCTATTA                                                944
Unigene0033916  TCCGGGTGCTTCCTCCCGCGAGACAAAGCTTTGGCTTTGTATTA                                                944
```

iPR amino acid sequences alignment

```
MpiPR           MAEVQRYALVTGANKGIGFEICRQLAEKGIIVILTSRNEKRGLEARQKLLKELNVSENRLVFHQLDVTDLASVAAVAVFIKSKFGKLDIL   90
Unigene0033916  MANVQRYALVTGANKGIGFEICRQLAEKGIIVILTARNEKRGIEAHQRLLKELNISKNHLVFHQLDVTDPASIAAVAVFIKSTFGKLDIL   90

MpiPR           VNNAGVSGVEMVGDVSVFNEYIEADFKALQALEAGAKEEPPFKPKANGEMIEKFEGAKDCVVTNYYGPKRLTQALIPLLQLSPSPRIVNV  180
Unigene0033916  VNNAGVSGVEMVGDVSVFNEYIEADFNALQALEAGAKEEPPFKPKANGEMIEKFEGAKDCVETNYYGPKRLTQALIPLLQLSPSPRIVNV  180

MpiPR           SSSFGSLLLLWNEWAKGVLGDEDRLTEERVDEVVEVFLKDIKEGKLEESQWPPHFAAERVSKAALNAYTKIAAKKYPSFRINAICPGYAK  270
Unigene0033916  SSSFGSLLLLWNEWAKGVLGDEDSLTEERVDEVVEVFLKDIKEGKLEENQWPPHFAAERVSKAALNAYTKIAAKKYPSFRINAICPGYAK  270

MpiPR           TDITFHAGPLSVAEAAQVPVKLALLPDGGPSGCFFPRDKALAL                                                 313
Unigene0033916  TDITFHAGPLSVSEAAQVPVKLALLPDGGPSGCFLPRDKALAL                                                 313
```

MFS nucleotide sequences alignment

```
MpMFS           ATGGCCGCTCTTCTAGTATTTTTCTCTGTCTCTTTAATCTTACTGGCGGTCCTTTTCCATAAGCGAAAGTCCAGTCTTT...CCTCAAGA   87
Unigene0030907  ATGGCAGCTCTTCTAGTATTTTTCTCTTTCTCTTTAATCTTGCTGGCGGTCCTTTTCCATAAACGAAGCTCCAGTCTTCTTCCCTCAAGA   90

MpMFS           AAGAGGC...CGCCGCCGTCTCCATTAAGGCTTCCGGTGATCGGCCATTTCCACCTGATCGGAGCCCTCTCCCACCGCTCCTTCACCTCC   174
Unigene0030907  AAGAGGCGGCTGCCGCCGTCTCCATCAAGGCTTCCGGTGATCGGCCATTTGCACCTGATCGGATCCCTCGCCCACCGCTCCTTCCACTCC   180

MpMFS           TTATCCAAGCGCTACGGCGAGGTGATGCTACTCCATTTCGGCAGCGCTCCTGTCCTAGTGGCCTCCTCAGCGGCGGCGGCGCGTGAGATC   264
Unigene0030907  TTATCCAAGCGCTACGGCGAGGTGATGCTACTCCATTTCGGCAGCGCTCCGGTCCTCGTGGCCTCCTCAGCCGCGGCGGCGCGTGAGATC   270

MpMFS           ATGAAGAACCAAGACGTGATCTTCGCGAGCAGGCCGAGGCTGAGCATCTTCGACAGGCTGATGTACAGCGGCAAGGGCGTGGCCTTCGCC   354
Unigene0030907  ATGAAGAACCAAGACATGATCTTCGCGAGCAGGCCGAGGCTGAGCATCTCCGACAGGCTGCTGTACAGTGGCAAGGACGTGGCCTTCGCC   360

MpMFS           CCCTACGGCGAGCACTGGCGCAACGCGCGGAGCATGTGCATGCTGCAGCTGCTCAGCGCTAAGAGGGTCCAGTCGTTCGGCGGATTCGA   444
Unigene0030907  GCCTACGGCGAGCACTGGCGCCACGCGCGGAGCATGTGCGTGCTGCAGCTGCTCAGCGCTAAGAGGGTCCAGTCGTTCCGTCGGATCCGA   450

MpMFS           GAAGAGGAAACGTCGGCTATGATTGAGAAGATCAGGCGATCGAAGCCGACGACGGTCGTGAACTTGAGCGAGATGTTCATGGCGCTGACG   534
Unigene0030907  GAAGAGGAAACGTCGGCGATGATTGAGAAGATCAGGCGATCGCAGCCGT...CGGTCGTGAACTTGAGCGAGATGTTCATGGCGCTGACG   537

MpMFS           AACGGCGTAATTCACAGGGCGGTGTTAGGGCGGAAGGGCGACGGCGGAGATGATTTTAATCGGATTCTGATCAAGGTTATCAAGCTCTTG   624
Unigene0030907  AACGGCGTAGTTCACAGGGCGGCGTTAGGGCGGAAGGACGGCGGCGGAGATGATTCTAATCTGACTTTGAACAAGTTTATCGAGCTCTTG   627

MpMFS           GGAAGTTTCAACGTGGGGGATTACGTTCCATGGCTGTCTTGGATCAACCGGATAAATGGCGTCGATGCAGAAGTGGAAAAAGTCGGTACA   714
Unigene0030907  GGACGTTTCAACGTGGGGGATTACGTTCCATGGCTGGCTTGGATTAACCGGATTAATGGCGTCGATGCAGAAGTGGAAAAAGTCTTTAGA   717

MpMFS           AAGTTGGATGGATCAATGGAGGGTATTCTTCGAAAATATAGAAGGAAGAAGGTGGGTGATGATGAAACGAATTTCGTAGACACATTGCTT   804
Unigene0030907  AAGTTGGATGGATTTATAGAGGGTATTCTTCGAGAATATAGAATGAAGAATGATACACATGCATCAACGAATTTCGTAGACACGTTGCTT   807

MpMFS           CAGTTTCAGAGAGAGAGTAAAGATACTGACCCCGTCGAGGATGACGTAATCAAAGCTCTAATCTTCGACATGGTTTCTGCCGGAACCGAT   894
Unigene0030907  CAGTTTCAGAGAGAGAGTAAAGATACTAACCCCGTCGAGGATGACGTAATCAAAGCTCTAATCCTCGACATGTTTGCCGCCGGAATCGAT   897

MpMFS           ACGACTTTCGCGGCTCTAGAGTGGACGATGGCGGAGCTCATAAAAAACCCGCGTACCCTAAAAACTTTGCAGAACGAGGTGCGAGAAGTT   984
Unigene0030907  ACGACGTCCGCGGTTCTAGAGTGGACGATGGCGGAGCTCATAAGAAATCCGCGAACCCTAAAAACTCTGCAGAACGAGGTGCGAGAAGTT   987

MpMFS           TCTAGAAACAAAGGTGGGATTACCGAGGATGACGTGGACAAGATGCCATATCTGAAAGCAGTATCCAAGGAGATTCTACGCCTACATCCA   1074
Unigene0030907  TCTAGAAACAAAGGCGGGATTACTGAGGATGACGTGGACAAGATGCCATACCTGAAAGCAGTATCCATGGAGATTCTACGCCTACATCCA   1077

MpMFS           CCTTTCGCAATTCTGCTCCCTCGAGAATTGACTCAAGACGCCAATATGCTCGGCTACGACATCCCACGTGGCACGGTCGTGTTGGTCAAC   1164
Unigene0030907  CCTTTCCCATCGCTGCTCCCTCGAGAATTGACTCAAGACGCCAACATGCTCGGCTACGACGTCCCACGTGGCACGCTCGTGTTGGTCAAC   1167

MpMFS           AACTGGGCCATATCGAGAGACCCCTCGTTGTGGGAAAATCCCGAAGAATTTCGTCCAGAAAGGTTCCTCGAGACGAGCATAGACTATAAA   1254
Unigene0030907  AACTGGGCCATATCGAGAGACCCCTCGTTGTGGGAAAATCCCGAAGAATTTCGTCCAGAAAGGTTCCTCGAGACGAGCATAGACTATAAA   1257

MpMFS           GGGTTGCATTTTGAGATGCTTCCGTTCGGGTCGGGTCGAAGAGGGTGCCCCGGATCCACGTTTGCGATGGCTTTATACGAGCTTGCACTA   1344
Unigene0030907  GGGATGCATTTCGAGATGCTTCCGTTCGGGTCGGGACGAAGAGGGTGCCCCGGAATCACGTTTGCGATGTCTTTATACGAGCTTGCACTA   1347

MpMFS           TCCAAGCTGGTAAACGAGTTCGATTTCAGATTGGGTAATGGAGATAGAGCGGAGGATTTGGACATGACTGAAGCTCCTGGATTTGTAGTC   1434
Unigene0030907  TCCAAGTTAGTAAACGAGTTCGATTTGAGATTGGCTAATGGAGATAGAGTGGAGGATTTGGACA.........................   1411

MpMFS           CATAAGAAGTCTCCTTTGCTTGTGCTTGCTACTCCACGTCAATCTTG                                            1481
Unigene0030907  ...............................................                                            1411
```

MFS amino acid sequences alignment

```
MpMFS           MAALLVFFSVSLILLAVLFHKRKSSLSS..RKRPPPSPLRLPVIGHFHLIGALSHRSFTSLSKRYGEVMLLHFGSAPVLVASSAAAAREI   88
Unigene0030907  MAALLVFFSFSLILLAVLFHKRSSSLLPSRKRRLPPSPSRLPVIGHLHLIGSLAHRSFHSLSKRYGEVMLLHFGSAPVLVASSAAAAREI   90

MpMFS           MKNQDVIFASRPRLSIFDRLMYSGKGVAFAPYGEHWRNARSMCMLQLLSAKRVQSFGGIREEETSAMIEKIRRSKPTTVVNLSEMFMALT   178
Unigene0030907  MKNQDMIFASRPRLSISDRLLYSGKDVAFAAYGEHWRHARSMCVLQLLSAKRVQSFRRIREEETSAMIEKIRRSQPS.VVNLSEMFMALT   179

MpMFS           NGVIHRAVLGRKGDGGDDFNRILIKVIKLLGSFNVGDYVPWLSWINRINGVDAEVEKVGTKLDGSMEGILRKYRRKKVGDDETNFVDTLL   268
Unigene0030907  NGVVHRAALGRKDGGGDDSNLTLNKFIELLGRFNVGDYVPWLAWINRINGVDAEVEKVFRKLDGFIEGILREYRMKNDTHASTNFVDTLL   269

MpMFS           QFQRESKDTDPVEDDVIKALIFDMVSAGTDTTFAALEWTMAELIKNPRTLKTLQNEVREVSRNKGGITEDDVDKMPYLKAVSKEILRLHP   358
Unigene0030907  QFQRESKDTNPVEDDVIKALILDMFAAGIDTTSAVLEWTMAELIRNPRTLKTLQNEVREVSRNKGGITEDDVDKMPYLKAVSMEILRLHP   359

MpMFS           PFAILLPRELTQDANMLGYDIPRGTVVLVNNWAISRDPSLWENPEEFRPERFLETSIDYKGLHFEMLPFGSGRRGCPGSTFAMALYELAL   448
Unigene0030907  PFPSLLPRELTQDANMLGYDVPRGTLVLVNNWAISRDPSLWENPEEFRPERFLETSIDYKGMHFEMLPFGSGRRGCPGITFAMSLYELAL   449

MpMFS           SKLVNEFDFRLGNGDRAEDLDMTEAPGFVVHKKSPLLVLATPRQ                                               492
Unigene0030907  SKLVNEFDLRLANGDRVEDLD.......................                                               470
```

PR nucleotide sequences alignment

```
MpPR            ATGGTGATGAACAAGCAAATTGTACTCAACAACTACATTAATGGTTCTCTAAAACAATCCGACTTGGCGTTGAGAACTTCCACGATCTGC    90
Unigene0047772  ATGGTGATGAACAAGCAAATTGTACTCAACAACTACGTCAACGGTTCTTTAAAAGAATCCGACTTGTCGTTGAGAACTTCCACGATCTGC    90

MpPR            ATGGAGATCCCAGATGGCTGCAACGGTGCCATTTTGGTCAAGAACTTGTACTTGTCCGTCAATCCTTATCTCATTCTTCGCATGGGAAAA   180
Unigene0047772  ATGGAGATCCCACATGGCTGCAACGGTGCCGTTTTGGTCAAGAACTTGTACTTGTCCGTCAATCCTTATCTCATTCTTCGCATGGGAAAA   180

MpPR            CTCGATATCCCACAGTTTGATTCCATCCTTCCTGGCTCTACTATTGTTAGCTATGGAGTGTCAAAAGTATTGGATTCGACGCATCCGAGT   270
Unigene0047772  CTCGATATCCCACAGTTTGATTCCATCCTTCCTGGCTCTACTATTGTTAGCTATGGAGTGTCAAAAGTATTGGATTCGACGCATCCGAGT   270

MpPR            TACGAGAAAGGCGAACTGATTTGGGGGTCACAAGCTGGATGGGAGGAATATACCCTTATCCAAAATCCATATAATTTGTTTAAAATCCAA   360
Unigene0047772  TACGAGAAAGGCGAACTGATTTGGGGGTCACAAGCTGGATGGGAGGAATATACCCTTATCCAAAATCCATATAATTTGTTTAAAATCCAA   360

MpPR            GACAAAGATGTGCCTTTATCCTACTATGTTGGGATTCTAGGAATGCCTGGGATGACAGCATATGCAGGATTTTTTGAGATTTGCTCTCCG   450
Unigene0047772  GACAAAGATGTGCCTTTATCCTACTATGTTGGGATTCTAGGAATGCCTGGGATGACAGCATATGCAGGATTTTTTGAGATTTGCTCTCCG   450

MpPR            AAAAAAGGCGAAACTGTGTTTGTAACGGCTGCAGCAGGATCTGTGGGCCAGCTTGTTGGTCAGTTTGCAAAGATGTTTGGGTGCTATGTT   540
Unigene0047772  AAAAAAGGCGAAACTGTGTTTGTAACGGCTGCAGCAGGATCTGTGGGCCAGCTTGTTGGTCAGTTTGCAAAGATGTTTGGGTGCTATGTT   540

MpPR            GTTGGAAGTGCAGGGAGCAAAGAGAAGGTTGATCTTTTGAAGAACAAATTTGGGTTCGATGATGCATTTAATTATAAAGAAGAGAGTGAT   630
Unigene0047772  GTTGGAAGTGCAGGGAGCAAAGAGAAGGTTGATCTTTTGAAGAACAAATTTGGGTTCGATGATGCATTTAATTATAAAGAAGAGAGTGAT   630

MpPR            TATGATACTGCTTTGAAGAGGCACTTCCCCGAAGGAATTGATATATACTTCGATAATGTTGGAGGGAAGATGCTTGAAGCTGTGATCAAC   720
Unigene0047772  TATGATACTGCTTTGAAGAGGCACTTCCCCGAAGGAATTGATATATACTTCGATAATGTTGGAGGGAAGATGCTTGAAGCTGTGATCAAC   720

MpPR            AACATGAGAGTCCACGGCCGCATCGCGGTATGTGGGATGGTCTCCCAGTACAGCCTGAAGCAGCCTGAAGGCGTCCACAACTTGCTTAAG   810
Unigene0047772  AACATGAGAGTCCACGGCCGCATCGCGGTATGTGGGATGGTCTCCCAGTATAGCCTGAAGCAGCCCGAAGGCGTCCACAACTTGCTTAAG   810

MpPR            CTAATCCCAAAGCAAATTCGTATGCAAGGGTTTGTTGTTGTTGATTACTATCATCTCTACCCAAAGTTCCTTGAGATGGTTCTGCCGCGC   900
Unigene0047772  CTCGTCCCAAAACAAATTCGTATGCAAGGGTTTGTCGTTGTTGATTACTATCATCTCTACCCAAAGTTCCTTGAAATGGTTCTGCCTTGC   900

MpPR            ATCAAGGAAGGAAAAGTGACATACGTCGAAGACATATCTGAAGGCCTTGAGAGCGCGCCTAGCGCTCTCTTGGGGGTGTACGTCGGTCGT   990
Unigene0047772  ATCAAGGAAGGAAAAGTGATATACGTCGAAGACATATCTGAAGGCCTTGAGAGTGCGCCTAGTGCTCTCTTGGGGGTGTATGTCGGTCGT   990

MpPR            AACGTTGGCAATCAAGTTGTTGCCGTTTCTCGCGAGTA                                                    1028
Unigene0047772  AACATTGGCAATCAGGTTGTTGCCGTTTCTCGCGAGTA                                                    1028
```

PR amino acid sequences alignment

```
MpPR            MVMNKQIVLNNYINGSLKQSDLALRTSTICMEIPDGCNGAILVKNLYLSVNPYLILRMGKLDIPQFDSILPGSTIVSYGVSKVLDSTHPS    90
Unigene0047772  MVMNKQIVLNNYVNGSLKESDLSLRTSTICMEIPHGCNGAVLVKNLYLSVNPYLILRMGKLDIPQFDSILPGSTIVSYGVSKVLDSTHPS    90

MpPR            YEKGELIWGSQAGWEEYTLIQNPYNLFKIQDKDVPLSYYVGILGMPGMTAYAGFFEICSPKKGETVFVTAAAGSVGQLVGQFAKMFGCYV   180
Unigene0047772  YEKGELIWGSQAGWEEYTLIQNPYNLFKIQDKDVPLSYYVGILGMPGMTAYAGFFEICSPKKGETVFVTAAAGSVGQLVGQFAKMFGCYV   180

MpPR            VGSAGSKEKVDLLKNKFGFDDAFNYKEESDYDTALKRHFPEGIDIYFDNVGGKMLEAVINNMRVHGRIAVCGMVSQYSLKQPEGVHNLLK   270
Unigene0047772  VGSAGSKEKVDLLKNKFGFDDAFNYKEESDYDTALKRHFPEGIDIYFDNVGGKMLEAVINNMRVHGRIAVCGMVSQYSLKQPEGVHNLLK   270

MpPR            LIPKQIRMQGFVVVDYYHLYPKFLEMVLPRIKEGKVTYVEDISEGLESAPSALLGVYVGRNVGNQVVAVSR                      341
Unigene0047772  LVPKQIRMQGFVVVDYYHLYPKFLEMVLPCIKEGKVIYVEDISEGLESAPSALLGVYVGRNIGNQVVAVSR                      341
```

MR nucleotide sequences alignment

```
MpMR            ATGGCAGATACGTTTACCCAAAGGTATGCTTTGGTTACTGGTGCAAACAAAGGAATCGGGTTCGAGATATGCAGGCAGTTAGCTTCGAAA   90
Unigene0041050  ATGGCAGATACTTTTACACAGAGGTATGCTTTGATTACCGGTGCAAACAAAGGAATCGGGTTCGAGATATGCAGGCAGTTAGCTTCGAAA   90

MpMR            GGAATGAAGGTGATTTTAGCTTCAAGAAACGAGAAGAGAGGCATCGAAGCTCGAGAAAGGCTACTTAAGGAGTCGAGATCAATTTCTGAT  180
Unigene0041050  GGAATGAAGGTGATTTTAGCTTCAAGAAACGAGAAGAGAGGCATCGAAGCTCGAGAAAGGCTACTTAAGGAATCGAGATCAATTTCTGAT  180

MpMR            GACGATGTTGTTTTTCATCAACTCGATGTTGCTGACCCTGCTAGCGCTGTTGCTGTTGCTCACTTCATCGAAACCAAATTCGGGAGGCTT  270
Unigene0041050  GACGATGTTGTTTTTCATCAACTTGATGTTGTTGACCCTGCTAGCGCTGTTGCTGTTGCTCACTTCATCGAAACCAAATTCGGGAGGCTT  270

MpMR            GATATTCTGGTGAATAATGCGGGATTTACTGGAGTAGCGATAGAGGGAGATATTTCAGTGTATCAAGAGTGTCTTGAGGCAAACATTATT  360
Unigene0041050  GATATTCTGGTGAATAATGCGGGATTTACTGGAGTAGCGATAGAGGGAGATATTTCAGTGTATCAAGAGTGTATTGAGGCAAACATTATT  360

MpMR            GCAGCTCAGGGTGGACAGGCACATCCATTCCATCCCAAAACTACTGGTAGGCTGATTGAGACATTGGAGGGTTCAAAAGAGTGTATAGAA  450
Unigene0041050  GCAGCTCAGAGTGGACAGGCACGTCCATTCCATCCCAAATCAAATGGTAGGCTTATTGAAACATTGGAGGGTTCAAAAGAGTGTATAGAA  450

MpMR            ACAAACTACTATGGCACAAAAAGAATAACAGAAACCCTAATTCCTCTCCTGCAAAAATCTGATTCACCAACAATTGTCAATGTCTCCTCC  540
Unigene0041050  ACAAACTACTATGGCACAAAAAGAATAACAGAAACCCTAATTCCTCTCCTGCAAAAATCTGATTCGCCAACAATTGTCAATGTCTCATCC  540

MpMR            ACCTTCTCGACTTTACTGCTCCAGCCTAATGAATGGGCAAAAGGAGTGTTTAGCAGCAA......CAGCCTGAATGAAGGGAAGGTGGAG  624
Unigene0041050  ACCTTCTCCACTTTACTGCTCCAGCCTAATGAATGGGCAAAAGGAGTATTTAGCAGTGAAGACACCAGTCTGAATGAAGAGAAGGTGGAG  630

MpMR            GAAGTTTTGCATGAATTTCTCAAGGATTTCATTGATGGTAAATTGCAACAAAACCACTGGCCTCCTAACTTTGCAGCCTACAAAGTATCG  714
Unigene0041050  GAAGTTTTGCATGAATTTATGAAGGATTTCATCGATGGTAAACTGCAACAAAACCACTGGCCTCCTAACCTTGCAGCCTACAAAGTGTCG  720

MpMR            AAAGCTGCTGTTAATGCGTATACTCGGATCATAGCGCGAAAGTACCCGAGTTTCTGCATAAATTCAGTGTGTCCTGGTTTTGTTAGAACA  804
Unigene0041050  AAAGCTGCTGTTAATGCATATACTCGGATCATAGCGCGAAAGTACCCGAGTTTCTGCATAAATTCAGTGTGTCCTGGTTTTGTTAGAACA  810

MpMR            GATATTTGCTACAATCTTGGAGTACTAAGTGAAGCTGAAGGTGCTGAAGCTCCGGTGAAGCTGGCTTTGTTGCCCGATGGCGGGCCCTCG  894
Unigene0041050  GATATTTGCTACAATCTTGGAGTACTAAGTGAAGCTGAAGGTGCTGAAGCTCCGGTGAAGCTGGCTTTGTTGCCCAATGGCGGGCCCTCG  900

MpMR            GGCTCCTTTTTCTCTCGAGAGGAAGCCTTGTCCTTGTACTA                                                  935
Unigene0041050  GGCTCCTTCTTCTCTCGAGAGGAAGCCTTGTCCTTGTACTA                                                  941
```

MR amino acid sequences alignment

```
MpMR            MADTFTQRYALVTGANKGIGFEICRQLASKGMKVILASRNEKRGIEARERLLKESRSISDDDVVFHQLDVADPASAVAVAHFIETKFGRL   90
Unigene0041050  MADTFTQRYALITGANKGIGFEICRQLASKGMKVILASRNEKRGIEARERLLKESRSISDDDVVFHQLDVVDPASAVAVAHFIETKFGRL   90

MpMR            DILVNNAGFTGVAIEGDISVYQECLEANIIAAQGGQAHPFHPKTTGRLIETLEGSKECIETNYYGTKRITETLIPLLQKSDSPTIVNVSS  180
Unigene0041050  DILVNNAGFTGVAIEGDISVYQECIEANIIAAQSGQARPFHPKSNGRLIETLEGSKECIETNYYGTKRITETLIPLLQKSDSPTIVNVSS  180

MpMR            TFSTLLLQPNEWAKGVFSSN..SLNEGKVEEVLHEFLKDFIDGKLQQNHWPPNFAAYKVSKAAVNAYTRIIARKYPSFCINSVCPGFVRT  268
Unigene0041050  TFSTLLLQPNEWAKGVFSSEDTSLNEEKVEEVLHEFMKDFIDGKLQQNHWPPNLAAYKVSKAAVNAYTRIIARKYPSFCINSVCPGFVRT  270

MpMR            DICYNLGVLSEAEGAEAPVKLALLPDGGPSGSFFSREEALSL                                                 310
Unigene0041050  DICYNLGVLSEAEGAEAPVKLALLPNGGPSGSFFSREEALSL                                                 312
```

Supplementary Figure 6 Alignment of nucleotide and amino acid sequences of 9 menthol biosynthetic genes from M. *piperita* and *M. canadensis*.
