# Supplementary material for: Transcriptome Analysis of JA Signal Transduction, Transcription Factors, and Monoterpene Biosynthesis Pathway in Response to Methyl Jasmonate Elicitation in Mentha canadensis L
Source: Int J Mol Sci. 2018 Aug 10;19(8):2364. doi: 10.3390/ijms19082364 (PMC6121529; doi:10.3390/ijms19082364)
Supplement: Supplementary file 1 [file ijms-19-02364-s001.zip › SupplementaryFiles/Supplementary_Figure_7.pdf]

A

```
MsYABBY5         ATGGATATGGCTGAGCAGCTGTGTTACATTCCTTGCAGCTACTGCAATATTATTCTTGCGGTAAGTGTTCCATGC    75
Unigene0062650   ATGGATATGGCTGAGCAGCTGTGTTACATTCCTTGCAGCTACTGCAATATTATTCTTGCGGTAAGTGTTCCATGC    75

MsYABBY5         AGCAGCTTGTTTGATGTAGTGACAGTTAGGTGTGGGCACTGCGCCAATCTTTGGACTGTGAATATGGCTGCTGCC   150
Unigene0062650   AGCAGCTTGTTTGATGTAGTGACAGTTAGGTGTGGGCACTGCGCCAATCTTTGGACTGTGAATATGGCTGCTGCC   150

MsYABBY5         TTCCCCTCTCTGCACGCCTCCTCCTTCCAAGATCTTCATCACCATCACCATCAGGGTCTTAGCTACGCTCCATCG   225
Unigene0062650   TTCCCCTCTCTGCACGCCTCCTCCTTCCAAGATCTTCATCACCATCACCATCAGGGTTTTAGCTACGCTCCATCG   225

MsYABBY5         GATTACAGAGTCGACCTCGGCTCCTCTTCCAAATGGAACTACAGGATGCCAATGCAGCCTCCTAGCTTCATCAAC   300
Unigene0062650   GATTACAGAGTCGACCTTGGCTCCTCTTCCAAATGGAACTACAGGATGCCAATGCAGCCTCCTAGCTTCATCAAC   300

MsYABBY5         AAACCAGATCAGAGAATCATCAATCGTCCCCCTGAGAAGCGGCAGCGTGTTCCATCTGCATACAATCAGTTCATT   375
Unigene0062650   AAACCAGATCAGAGAATCATCAATCGTCCCCCTGAGAAGCGGCAGCGTGTTCCATCTGCATACAATCAGTTCATT   375

MsYABBY5         AAGGAGGAAATTCAAAGAATCAAGGCCAACAATCCTGATATCAGCCATAGGGAAGCTTTCAGCACTGCTGCCAAA   450
Unigene0062650   AAGGAGGAAATTCAAAGAATCAAGGCCAACAATCCTGATATCAGCCATAGGGAAGCTTTCAGCACTGCTGCCAAA   450

MsYABBY5         AATTGGGCACACTTTCCTCATATCCATTTTGGGCTCATGCTGGAGAGCAAGAACCAAGATAATAAACTTGAGGAG   525
Unigene0062650   AATTGGGCACACTTTCCTCATATCCATTTTGGGCTCATGCTGGAGAGCAAGAACCAAGATAATAAACTTGAGGAG   525

MsYABBY5         GATTCTGAGAAGCATCAAATGAGAAGGGCAGCCGTTCTGAACAAATG                               572
Unigene0062650   GATTCTGAGAAGCATCAAATGAGAAGGGCAGCCGTTCTGAACAAATG                               572
```

B

```
MsYABBY5         MDMAEQLCYIPCSYCNIILAVSVPCSSLFDVVTVRCGHCANLWTVNMAAAFPSLHASSFQDLHHHHHQGLSYAPS    75
Unigene0062650   MDMAEQLCYIPCSYCNIILAVSVPCSSLFDVVTVRCGHCANLWTVNMAAAFPSLHASSFQDLHHHHHQGFSYAPS    75

MsYABBY5         DYRVDLGSSSKWNYRMPMQPPSFINKPDQRIINRPPEKRQRVPSAYNQFIKEEIQRIKANNPDISHREAFSTAAK   150
Unigene0062650   DYRVDLGSSSKWNYRMPMQPPSFINKPDQRIINRPPEKRQRVPSAYNQFIKEEIQRIKANNPDISHREAFSTAAK   150

MsYABBY5         NWAHFPHIHFGLMLESKNQDNKLEEDSEKHQMRRAAVLN                                       189
Unigene0062650   NWAHFPHIHFGLMLESKNQDNKLEEDSEKHQMRRAAVLN                                       189
```

C

```
MsMYB            ATGGGAAGAGCGCCGTGCTGTGAGAAAGTTGGGTTGAAGAGAGGGAGATGGACTGCAGAAGAAGATGAAAAGCTC    75
Unigene0030793   ATGGGAAGAGCGCCGTGCTGTGAGAAAGTTGGGTTGAAGAGAGGGAGATGGACTGCAGAAGAAGATGAAAAGCTC    75

MsMYB            AGAAAATATATTCAGGAAAATGGTGAAGGCTGCTGGCGATCATTGCCCAAGAATGCAGGTTTACTTAGATGTGGA   150
Unigene0030793   AGAAAATATATTCAGGAAAATGGTGAAGGCTGCTGGCGATCATTGCCCAAGAATGCAGGTTTACTTAGATGTGGA   150

MsMYB            AAGAGTTGCAGACTGAGATGGATTAATTATTTGAGATCAGATGTGAAGAGAGGGAATATTTCTTCTCAAGAAGAA   225
Unigene0030793   AAGAGTTGCAGACTGAGATGGATTAATTATTTGAGATCTGATGTGAAGAGAGGGAATATTTCTTCTCAAGAAGAA   225

MsMYB            GAAATCATCATTAATCTCCATGCATCTATGGGCAACAGGTGGTCCCTGATCGCCGCGCACTTGCCGGGTAGAACA   300
Unigene0030793   GAAATCATCATTAATCTCCATGCATCTATGGGCAACAGGTGGTCCCTGATCGCCGCGCACTTGCCGGGTAGAACA   300

MsMYB            GACAATGAAATCAAAAATTACTGGAACTCCCATTTGAGCAGAAAATTCCACGGTTTCCGCC...CTAATCCACAG   372
Unigene0030793   GACAATGAAATCAAAAATTACTGGAACTCCCATTTGAGTAGAAAATTCCACGGTTTCCGCCGCCCTAATCCACAG   375

MsMYB            TTCATTCCGCCGCCGCCGCCTCCGCCGCCATCCTCCAAGCCCAAGAAGACAAAGAACAGTAATAAGAAGGCAAAG   447
Unigene0030793   TTCATTCCGCCGCCGCCGCCTCCGCAGCCGTCCTCCAAGCCCAAGAAGACAAAGAACAGTAATAAGAAGGCAAAG   450

MsMYB            GCGGCAGCTAAAACCGCCACTGCCGCCGTCGTCATGCCGACTACCCCCACTCCGGAGAAAGAGTCCTCGGTGGGC   522
Unigene0030793   GCGGCAGCTAAAACTGCCACTGCCGCCGTCGTCATGCCGACTACCCCCACTCCGGAGAAAGAGTCCTCGGTGGGC   525

MsMYB            AGACCTGGGAAGGAGAGAGAAAGTGAAGCGAGAGAGAGCGGCAGCTCCATGGTGGGAGAGTTGGACGATTTAAAT   597
Unigene0030793   AGACCTGGGAAGGAGAGCGAAAGTGAAGCGAGAGAGAGCGGCAGCTCCATGGTGGGAGAGTTGGACGATTTAAGT   600

MsMYB            ATGACGGAGGACTTGAGCGGCCTCTGGGGCCCCACACTGGACTTCGGGACCGGAAGTGAAATTTCGGATCCGGGT   672
Unigene0030793   ATGACGGAGGACTTGAGCGGCCTCTGGGGCCCCACACTGGACTTCGGGACCGGAAGTGAAATTTCGGATCCGGGT   675

MsMYB            CTGGGCCAGCTAGATAACTCGTCGTTGCAAATTTACGAAGAAACGCTTTCGTGGATTTGGAACGACGAGGACGAC   747
Unigene0030793   CTGGGCCAGCTAGATAACTCGTCGTTGCAAATTTATGAAGACACGCTTTCGTGGATTTGGGACGACGAGGACGAC   750

MsMYB            AAGTGGAATTCCAACGTGGACAACGGAGAAATGGATGGTGCAATGCTTTCTTGGTTGTTGTCATG             812
Unigene0030793   AAGTGGAATTCCAACGTGGCCAACGGAGAAATGGATGGTGCAATGCTTTCTTGGTTGTTGTCATG             815
```

D

```
MsMYB            MGRAPCCEKVGLKRGRWTAEEDEKLRKYIQENGEGCWRSLPKNAGLLRCGKSCRLRWINYLRSDVKRGNISSQEE    75
Unigene0030793   MGRAPCCEKVGLKRGRWTAEEDEKLRKYIQENGEGCWRSLPKNAGLLRCGKSCRLRWINYLRSDVKRGNISSQEE    75

MsMYB            EIIINLHASMGNRWSLIAAHLPGRTDNEIKNYWNSHLSRKFHGFR.PNPQFIPPPPPPPSSKPKKTKNSNKKAK   149
Unigene0030793   EIIINLHASMGNRWSLIAAHLPGRTDNEIKNYWNSHLSRKFHGFRRPNPQFIPPPPPPQPSSKPKKTKNSNKKAK   150

MsMYB            AAAKTATAAVVMPTTPTPEKESSVGRPGKERESEARESGSSMVGELDDLNMTEDLSGLWGPTLDFGTGSEISDPG   224
Unigene0030793   AAAKTATAAVVMPTTPTPEKESSVGRPGKESESEARESGSSMVGELDDLSMTEDLSGLWGPTLDFGTGSEISDPG   225

MsMYB            LGQLDNSSLQIYEETLSWIWNDEDDKWNSNVDNGEMDGAMLSWLL                                 269
Unigene0030793   LGQLDNSSLQIYEDTLSWIWDDEDDKWNSNVANGEMDGAMLSWLL                                 270
```

Supplementary Figure 7 Alignment of nucleotide and amino acid sequences of MsYABBY5 and MsMYB with their homologs in *M. canadensis*. A, B: nucleotides and amino acid sequences of MsYABBY5 and Unigene0062650; C, D: nucleotides and amino acid sequences of MsMYB and Unigene0030793.
