# Supplementary material for: Transcriptome Analysis of JA Signal Transduction, Transcription Factors, and Monoterpene Biosynthesis Pathway in Response to Methyl Jasmonate Elicitation in Mentha canadensis L
Source: Int J Mol Sci. 2018 Aug 10;19(8):2364. doi: 10.3390/ijms19082364 (PMC6121529; doi:10.3390/ijms19082364)
Supplement: Supplementary file 1 [file ijms-19-02364-s001.zip › SupplementaryFiles/Supplementary_Table_4.docx]

**Supplementary Table 4. Sequences of primers used for qRT-PCR**

| **Unigenes** | **Forward primers** | **Reverse primers** |
| --- | --- | --- |
| Unigene0052366 (JAZ) | 5’-GCCAGTATTTGAAGGAGAAGGG-3’ | 5’-TATGGTCATCGGCGCAGTC-3’ |
| Unigene0045221 (JAZ) | 5’-CAGGCAAAGTGTTGGTGTT-3’ | 5’-CCACAAGAAATCTGGGAGC-3’ |
| Unigene0040079 (JAZ) | 5’-GATCTAATAATGCCCTCCCT-3’ | 5’-TCACGCTCTTCACCACCT-3’ |
| Unigene0071645 (TPL) | 5’-TCCAGAGGAGGTCAACAA-3’ | 5’-GCAAGTCATCGGTAGAGTG-3’ |
| Unigene0069251 (MYC2) | 5’-TCGGCTCCTATGCCTAAT-3’ | 5’-ATCGGTCTGTATGTTGTCTGAAT-3’ |
| Unigene0001153 (MYC2) | 5’-GCGTGGTCAATCCAAATAC-3’ | 5’-TCTACCGAAGATACTGAAGCA-3’ |
| Unigene0069748 (MYC2) | 5’-GGACGAGTGTCAACAGAGG-3’ | 5’-ATCCAATCCGAAGGCATA-3’ |
| Unigene0058216 (NINJA) | 5’-CGGCTCATCTGGGATTTCT-3’ | 5’-GCCCGTTCTCCTTTGTCG-3’ |
| Unigene0011510 (NINJA) | 5’-GGAAAGGCGAGGAGGTCA-3’ | 5’-AAGGAAGAAGAGGAGTTAGGGTT-3’ |
| Unigene0005346 (NINJA) | 5’-CCTTGCGTCTCAACCACAG-3’ | 5’-CATGATGCTCACCTCCGATT-3’ |
| Unigene0070028 (NINJA) | 5’-AATGTTCGTCGTCAGTTTCA-3’ | 5’-AGCCAGTATCGGTGTTGC-3’ |
| Unigene0070029 (NINJA) | 5’-GGCGGTCCGAGAAACAGA-3’ | 5’-CTCCAAACTCAAACTCCCACTT-3’ |
| Unigene0045757(GPPS-l) | 5’-TACATCCACCACCACAAGACGG-3’ | 5’-TTCCTGAGCCTCGCCACCT-3’ |
| Unigene0038587 (GPPS-s) | 5’-CGTCCACGAACACCTCCCT-3’ | 5’-CCGGCCAGCAACTCAAAC-3’ |
| Unigene0018537 (LS) | 5’-CGACCAACTTCCCGATTA-3’ | 5’-AACCACCGTGCCTCTACC-3’ |
| Unigene0052571 (L3OH) | 5’-CCCGAAAGGTTTGACGATGT-3’ | 5’-CAGATTCTTCTTCCCGCTCC-3’ |
| Unigene0033747 (iPD) | 5’-GCGACATCACCGACGAGGAA-3’ | 5’-GCCCGCGTTGCAGAACAT-3’ |
| Unigene0033916 (iPR) | 5’-GTTGGAGATGTTTCCGTGTT-3’ | 5’-TGGACCATAGTAGTTTGTTTCG-3’ |
| Unigene0030907 (MFS) | 5’-AGAGGGTCCAGTCGTTCCG-3’ | 5’-ACTACGCCGTTCGTCAGC-3’ |
| Unigene0047772 (PR) | 5’-GCGTCCACAACTTGCTTA-3’ | 5’-TACGACCGACATACACCC-3’ |
| Unigene0041050 (MR) | 5’-ACTGGAGTAGCGATAGAGGG-3’ | 5’-GATTTGGGATGGAATGGAC-3’ |
| Actin | 5’-CCAGGAATTGCTGATAGGATGAG-3’ | 5’-GCGCCACCACCTTAATCTTC-3’ |
