# Supplementary figures and images for: Transcriptome Analysis of JA Signal Transduction, Transcription Factors, and Monoterpene Biosynthesis Pathway in Response to Methyl Jasmonate Elicitation in Mentha canadensis L
Source: Int J Mol Sci. 2018 Aug 10;19(8):2364. doi: 10.3390/ijms19082364 (PMC6121529; doi:10.3390/ijms19082364)

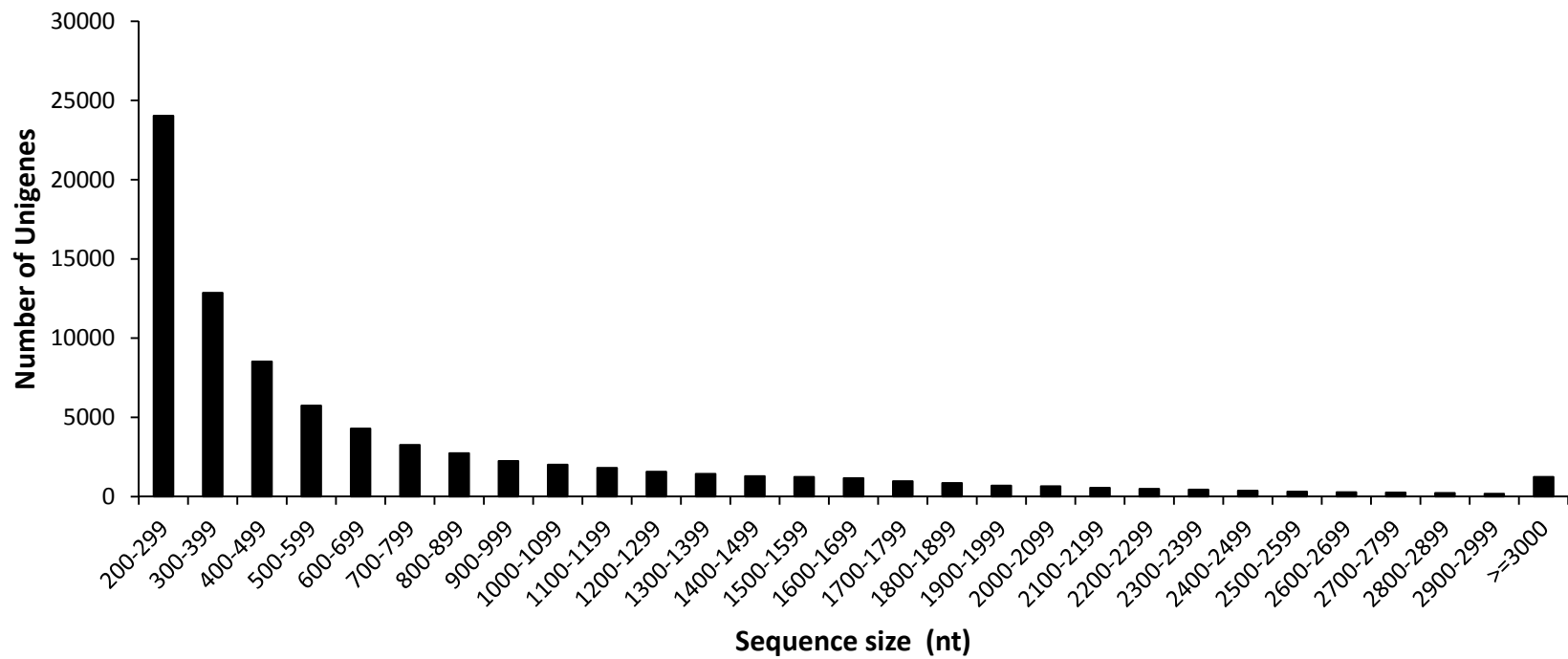

**Supplementary Figure 1 Length distribution of assembled unigenes.**

Supplement: Supplementary file 1 [file ijms-19-02364-s001.zip › SupplementaryFiles/Supplementary_Figure_1.pdf]

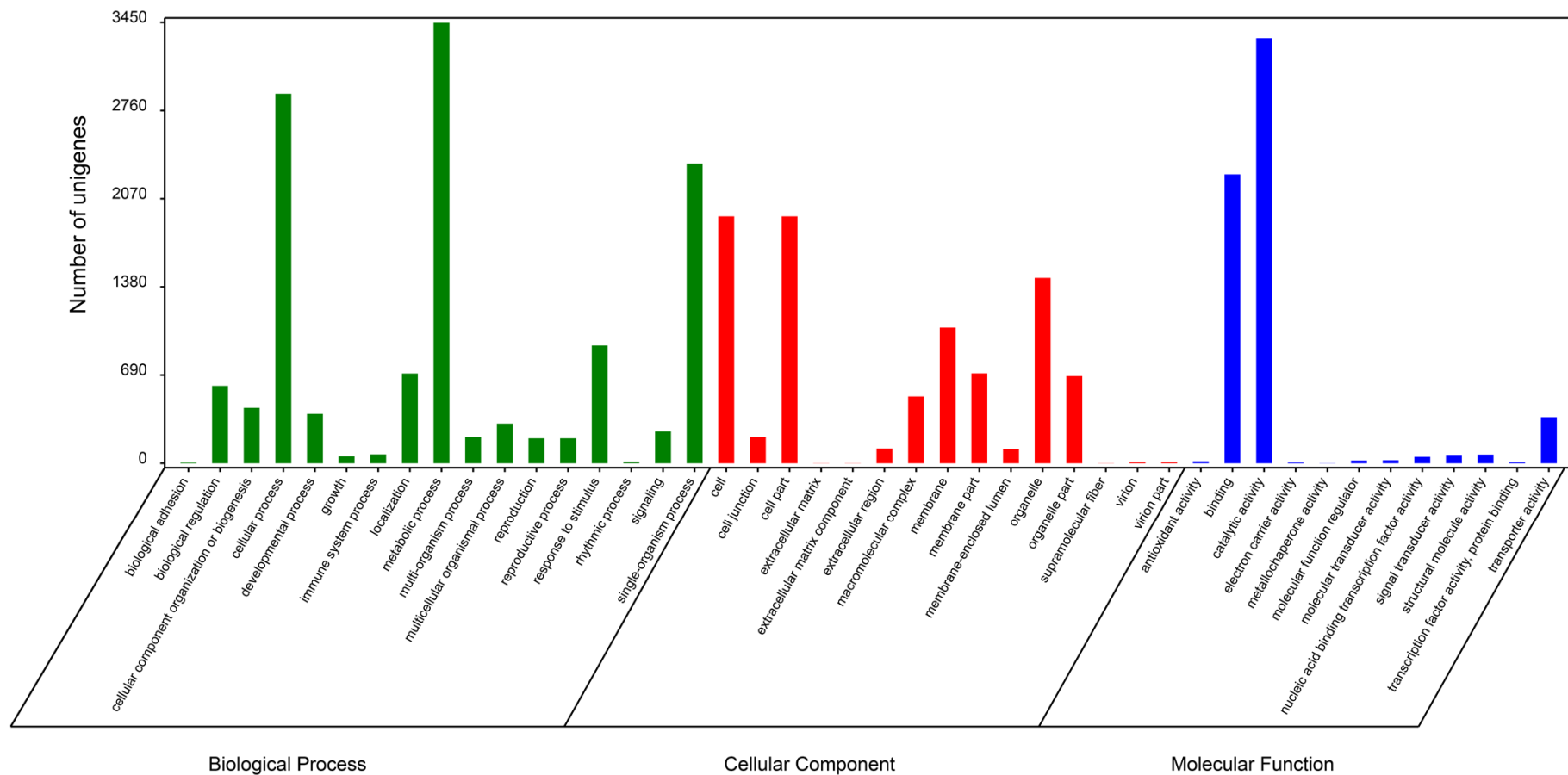

**Supplementary Figure 2 Gene ontology (GO) annotation of assembled unigenes.**

Supplement: Supplementary file 1 [file ijms-19-02364-s001.zip › SupplementaryFiles/Supplementary_Figure_2.pdf]

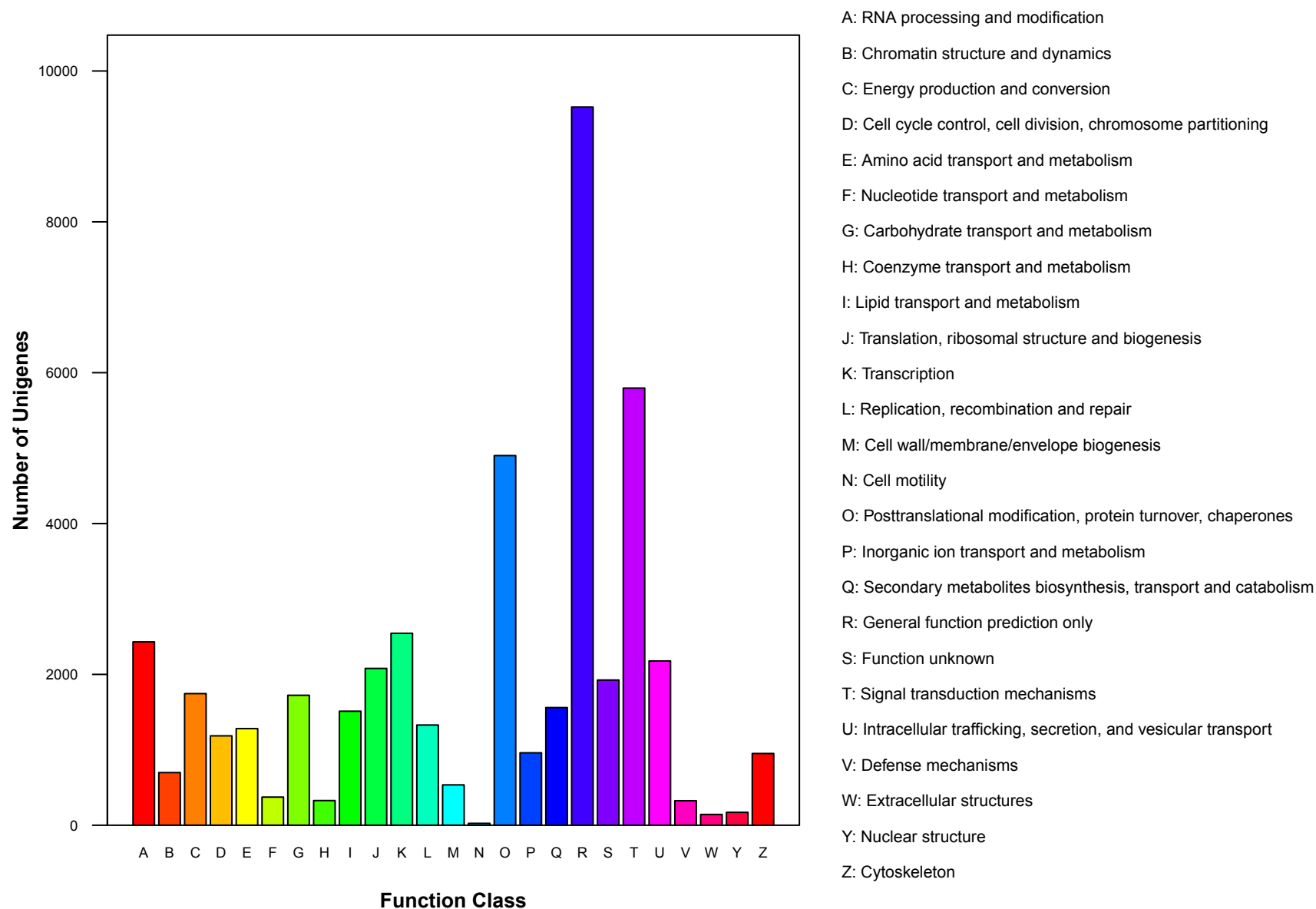

Supplementary Figure 3 KOG functional classification of assembled unigenes.

Supplement: Supplementary file 1 [file ijms-19-02364-s001.zip › SupplementaryFiles/Supplementary_Figure_3.pdf]

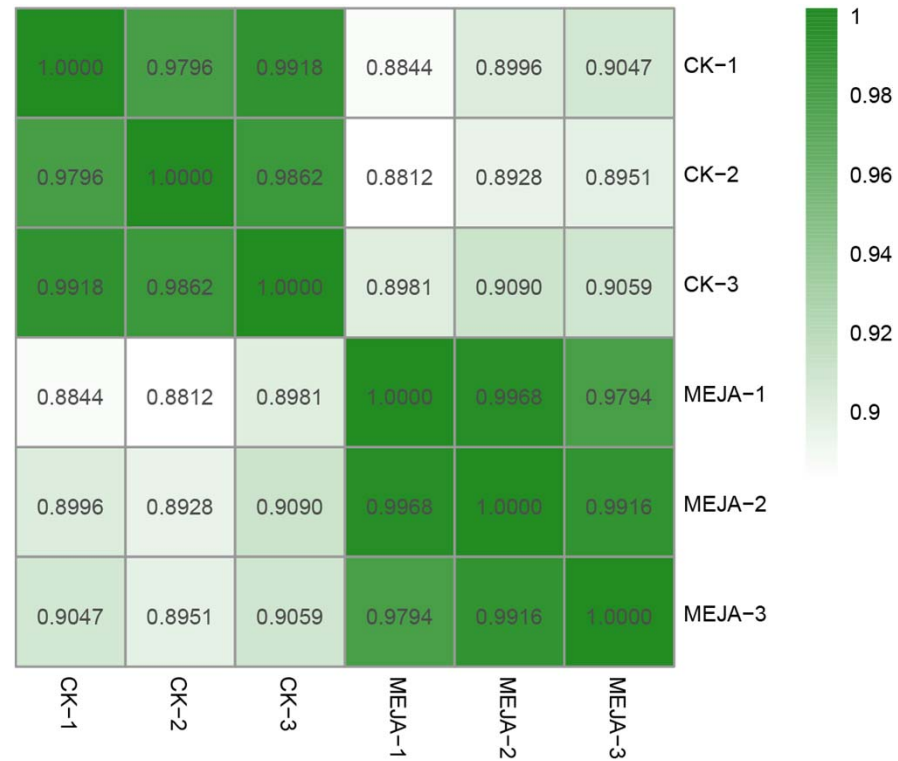

**Supplementary Figure 5 The pearson correlation between samples in RNA-seq.**

Supplement: Supplementary file 1 [file ijms-19-02364-s001.zip › SupplementaryFiles/Supplementary_Figure_5.pdf]
